# Correction: Healthcare-seeking behaviour of fever cases in Magude district, southern Mozambique: A qualitative study

**DOI:** 10.1371/journal.pone.0336533

**Published:** 2025-11-10

**Authors:** Carlos Eduardo Cuinhane, Julia Montaña Lopez, Hoticha Nhantumbo, Helder Djive, Ilda Murato, Beatriz Galatas, Caterina Guinovart, Francisco Saúte, Pedro Aide, Neusa Torres, Khátia Munguambe

There are errors in the author affiliations. The correct affiliations are as follows:

Carlos Eduardo Cuinhane^1,2^, Julia Montaña Lopez^2,3,4^, Hoticha Nhantumbo^2^, Helder Djive^2^, Ilda Murato^2^, Beatriz Galatas^2,3^, Caterina Guinovart^3^, Francisco Saúte^2^, Pedro Aide^5^, Neusa Torres^2,6,7^, Khátia Munguambe^2,8^

**1** Department of Sociology, Faculty of Arts and Social Sciences, Eduardo Mondlane University, Maputo, Mozambique, **2** Centro de Investigação em Saúde de Manhiça, Maputo, Mozambique, **3** ISGlobal, Hospital Clinic-Universitat de Barcelona, Barcelona, Spain, **4** Universitat de Barcelona (UB), Barcelona, Spain, **5** National Institute of Health, Ministry of Health, Maputo, Mozambique, **6** Department of Global Health and Social Medicine, King’s College London, London, United Kingdom, **7** SAMRC Developmental Pathways for Health Research Unit, School of Clinical Medicine, University of the Witwatersrand, Johannesburg, South Africa, **8** Faculty of Medicine, Eduardo Mondlane University, Maputo, Mozambique.
