# Supplementary material for: Healthcare-seeking behaviour of fever cases in Magude district, southern Mozambique: A qualitative study
Source: PLoS One. 2025 May 29;20(5):e0323117. doi: 10.1371/journal.pone.0323117 (PMC12122035; doi:10.1371/journal.pone.0323117)
Supplement: S1 Appendix — Semi-structured interviews (SSIs) guides and focus group discussions (FGD) guides used to collect qualitative data, translated from Portuguese into English. (DOCX) [file pone.0323117.s001.docx]

**S1 Appendix 1. Data collection tools-English version**

S1 Table 1. Semi-structured interview (SSI) guide for general population (community leaders, household heads, elders, women with decision-making power, women without decision-making power and pregnant women)

Interview data

| **Ref./File/Audio**  **(CARE-SOC-ESE-*NumESE-mmdd*)** | CARE-SOC\|__\|__\|-\|__\|__\|__\|__\| |
| --- | --- |
| **Date** | \|__\|__\|-\|__\|__\|-\|__\|__\|__\|__\| |
| **Specific place** |  |
| **Participant name initials** | \|__\|__\| |
| **Age of the participant** | \|__\|__\|__\| years |
| **Gender of the participant** | □ Male □ Female |
| **Origin** | □ Magude □ Gaza □ Inhambane □ Manhiça □ Moamba □ Maputo city □ Other district of Maputo province □ Rest of Mozambique □ South Africa □ Eswatine □ Other country of Africa □ Other country |
| **How long have you been living in Magude?** | \|__\|__\| months or \|__\|__\|__\| years |
| **Marital status** | □ Single □ Married □Marital Union □ Widower □Other (specify) ______________________ |
| **Number of children under your responsibility** | \|__\|__\|__\| |
| **Level of education** | □ None □ Primary □ Secondary □ Bachelor |
| **Ocuppation** | □ Housemaid □ Farmer □ Paid farmer □ Student □ Salesperson □ Services  □ Health worker □ Other (specify): ­____________________________________________ |
| **Type of employment** | □ formal work full time □ Formal work partial time □ Informal work (without contract) full time □ Informal work (without contract) partial time □ Sporadic work |
| **Main source of family incomes** | □ Incomes from formal work □ Incomes from informal work □ Agriculture □ Other (especify): ­______________ |
| **How many family members depends on this source of incomes?** | \|__\|__\|__\| |
| **Religion** | □Christian □Muslim □ Hindu □ Animist □ Atheist □ Other (esp): _______________________________ |
| **Posto Administrativo** | □ Magude-sede □ Motaze □ Mahele □ Panjane □ Mapulanguene |
| **Health facility used** | □ Magude-sede □ Chichuco □ Facazissa □ Motaze □ Moine □ Panjane □ Chicutso □ Mahele □ Captine □ Mapulanguene |
| **Distance to health facility** | \|__\|__\|__\| m/km |
| **Type of transport mostly used to health facility** | □ Walking □ Public transport □ Bicycle □ Motorized □ Personal car □ someone’ car □ Never go to health facility |
| **Time to reach to the health facility** | \|__\|__\| min or \|__\|__\| hours |
| **Time to reach community health worker** | \|__\|__\|__\| m/km |
| **Type of transport mostly used to community health worker** | □ Walking □ Public transport □ Bicycle □ Motorized □ Personal car □ Someone’s car □ Never go to community health worker |
| **Time to community health work** | \|__\|__\| min or \|__\|__\| hours |
| **Starting hour of SSI** | \|__\|__\|:\|__\|__\| |
| **Ending hour of SSI** | \|__\|__\|:\|__\|__\| |
| **Result of SSI** | □ Complete □Incomplete, Reasons: ________________________________________________  If applicable, reschedule the date: \|__\|__\|-\|__\|__\|-\|__\|__\|__\|__\| |

1. **CONTENT OF INTERVIEW**

| **Perceptions about worrying health conditions and the need to seek care in different ways**   - - - 1. How do you identify an abnormal or worrying health condition in yourself or a family member? What usually tells you that you or a family member is unwell?       2. How would you describe your general health status? (good, bad, normal)       3. Do you think there are some discomforts for which you need to visit a traditional doctor? Can these discomforts also be resolved in a Health Unit (US) or by community health worker (APE)?       4. For what types of discomfort do you seek medication directly from an informal drug seller? Why?       5. What types of discomfort do you think can be treated at home?       6. Have you ever heard community health workers (APEs) that exist in the community? For what types of discomfort do you go to an APE? For what types of discomforts do you take a child to an APE?       7. For what type of discomfort do you go to the Health Unit/Health Center? For what types of discomforts do you take a child to US/C Health Unit/Health Center S?       8. If you sometimes visit a traditional doctor, what makes you choose between seeking care from a traditional doctor and not a Health Unit/APE? And the other way around?   **Perceptions about fever and its causes**  1. Can you identify a fever? How does a fever normally present? What tells you that you or a family member has a fever?  2. What are the main causes of fevers that you know of?  a) If someone talks about malaria, ask about the cause of malaria and the other symptoms of malaria.  3. Are there fevers that need to be treated in different ways? If yes, give examples. Is it related to the cause of the fever?  a) If someone talks about malaria, ask what they do when they have malaria.  b) Are there fevers that can only be treated by traditional doctors? And only for APEs or in health facility? And only in health facility?  **Attitudes in cases of fever**  When you feel like you have a fever, or a family member feels like you have a fever:  1. Who do you ask for help first (e.g. another family member, APE, community leader, etc.) and what type of help do you initially seek (e.g. home care, medication, diagnosis, etc.)?  2. What makes you seek help outside your home? (e.g. recommendation from a family member, not being able to get out of bed, symptoms other than fever, identifying fever as malaria, etc.)  3. Who is the main person who makes the decision about what to do when you have a fever? What about when a child has a fever? At what time?  4. Who is the main person who cares for the sick at home?  5. What is the first form of help you seek outside your home (e.g. APE, health facility, traditional doctor, community leader, etc.)?  a) If not APE/health facility, what makes you finally go to an APE/health facility? (if anything makes you go to APE/health facility?  6. How long do you think it takes, in general, from the time you feel a fever until you make the decision to go to an APE/health facility? (if this decision is made), and how long does it take from when the decision is made to reaching an APE/health facility?  7. Do you have any preference between Health facility vs APEs for the treatment of fevers? If so, why?  8. If you have a family member who has been diagnosed with malaria, and when you return home, you find another family member with a fever, what would your reaction be?  **Barriers and facilitators of seeking care for fevers in the Health Unit or Health Center**  1. What are the main barriers/difficulties you encounter when seeking help at health facility when you or a family member has a fever? Note: there may be factors of your personal situation, factors related to health facility, general factors, others.  a) Do any of these difficulties prevent you from seeking help at a health facility?  2. What makes it easier for you to seek help at health facility when you or a family member has a fever?  **Barriers and facilitators of APE fever care-seeking**  3. What are the main barriers/difficulties you encounter when seeking help from APE when you or a family member has a fever?  a) Do any of these difficulties prevent you from seeking help from APE?  4. What makes it easier for you to seek help from APE when you or a family member has a fever?  5. Do you have support in your family or community to take care of other family members if you go to health facility or APE? If so, from whom do you receive support? (If the interviewee is a woman: is the husband at home normally?).  **Perspectives on the quality and cost of care in US / APEs**  1. When you arrive at health facility with a fever, what treatment do they give you? (Explore more, do they do TDR, or other exams?)  2. Overall, do you think you receive good quality care at health facility? If yes, why? If not, why not?  3. What about the APE? If yes, why? If not, why not?  4. What are the main costs involved in the process of curing a fever if you go to health facility? What about an APE? Are they affordable for you, or are they a deterrent to seeking healthcare?  5. Is cost a factor you consider before seeking health care? What do you do to cover the cost?  What are the factors that influence the decision-making to seek help at health facility/APE?  • Distance from health facility / APE  •Transport  • Opening hours  • Technical knowledge or training of health workers  • Waiting time for service  • Tools available at health facility /APE (diagnosis, medication)  • The way health workers treat people  • How often people at home get sick  • The number of people you will care for  • Have the support of your husband/wife (economically or on a practical level)  • Be familiar with the APE or health facility  • Total time you waste  **General recommendations**  1. In your opinion, what needs to be improved for the population to have easy access to health services? Or is it fine as it is?  Note:  If the participant has not identified malaria in the “Perceptions about fever and its causes” part, do the following questions: knowledge about malaria  2. In your opinion, when a person has fever, chills, headaches and sometimes vomiting, what illness should they have?  3. What do you know more about malaria?  4. If you mention another disease, explore further, ask if this disease is related to malaria?  •Cause  • Prevention/ Explore more about prevention  •Treatment  5. In your opinion, what could be the cause and what can be done to eliminate malaria? |
| --- |

**OBSERVATIONS: _________________________________________________________________________________________________**

**SIGNATURES**

NAME OF INTERVIEWR: ______________________________ SIGNATURE: _______________________ COD: |__|__|__|__|

S1 Table 2. Semi-structured interview (SSI) guide for travellers

**Interviewee data**

| **Ref./File/Audio**  **(CARE-SOC-ESE-*NumESE-mmdd*)** | CARE-SOC\|__\|__\|-\|__\|__\|__\|__\| |
| --- | --- |
| **Date** | \|__\|__\|-\|__\|__\|-\|__\|__\|__\|__\| |
| **Specific place** |  |
| **Participant name initials** | \|__\|__\| |
| **Age of the participant** | \|__\|__\|__\| years |
| **Gender of the participant** | □ Male □ Female |
| **Origin** | □ Magude □ Gaza □ Inhambane □ Manhiça □ Moamba □ Maputo city □ Other district of Maputo province □ Rest of Mozambique □ South Africa □ Eswatine □ Other country of Africa □ Other country of the world |
| **How long have you been living in Magude?** | \|__\|__\| months or \|__\|__\|__\| years |
| **Marital status** | □ Single □ Married □Marital Union □ Widower □Other (specify) ______________________ |
| **Number of children under your responsibility** | \|__\|__\|__\| |
| **Level of education** | □ None □ Primary □ Secondary □ Bachelor |
| **Occupation** | □ Housemaid □ Farmer □ Paid farmer □ Student □ Salesperson □ Services  □ Health worker □ Other (specify): ­____________________________________________ |
| **Type of employment** | □ formal work full time □ Formal work partial time □ Informal work (without contract) full time □ Informal work (without contract) partial time □ Sporadic work |
| **Main source of family incomes** | □ Incomes from formal work □ Incomes from informal work □ Agriculture □ Other (especify): ­______________ |
| **How many family members depends on this source of incomes?** | \|__\|__\|__\| |
| **Religion** | □Christian □Muslim □ Hindu □ Animist □ Atheist □ Other (esp): _______________________________ |
| **Posto Administrativo** | □ Magude-sede □ Motaze □ Mahele □ Panjane □ Mapulanguene |
| **Health facility used** | □ Magude-sede □ Chichuco □ Facazissa □ Motaze □ Moine □ Panjane □ Chicutso □ Mahele □ Captine □ Mapulanguene |
| **Distance to health facility** | \|__\|__\|__\| m/km |
| **Type of transport mostly used to health facility** | □ Walking □ Public transport □ Bicycle □ Motorized □ Personal car □ someone’ car □ Never go to health facility |
| **Time to reach to the health facility** | \|__\|__\| min or \|__\|__\| hours |
| **Time to reach community health worker** | \|__\|__\|__\| m/km |
| **Type of transport mostly used to community health worker** | □ Walking □ Public transport □ Bicycle □ Motorized □ Personal car □ Someone’s car □ Never go to community health worker |
| **Time to community health work** | \|__\|__\| min or \|__\|__\| hours |
| **Starting hour of SSI** | \|__\|__\|:\|__\|__\| |
| **Ending hour of SSI** | \|__\|__\|:\|__\|__\| |
| **Result of SSI** | □ Complete □Incomplete, Reasons: ________________________________________________  If applicable, reschedule the date: \|__\|__\|-\|__\|__\|-\|__\|__\|__\|__\| |

**Content of the interview**

| **Perceptions about health conditions of concern and the need to seek care in different ways**  1.How do you identify an abnormal or worrying state of health in yourself or a member of your family? What usually indicates to you that you are unwell?  2.How would you describe your general state of health (good, bad, normal)?  3.What kind of discomfort do you go to a traditional doctor for? For what kind of discomforts do you take a child to a traditional doctor? Can these discomforts also be solved in a health facility) or by a community health worker (APE)?  4.For what kind of discomforts do you seek medication directly from a drug dealer? Why would you do that?  5.For what kind of discomforts are you cared for at home?  6.Have you heard of multipurpose APEs that exist in the community? What kind of discomfort do you go to an APE for? What kind of discomfort do you take a child to an APE for?  7.For what kind of discomfort do you go to the Health Unit/Health Centre? For what kind of discomfort do you take a child to the health facility?  8.What makes you choose to seek care from a traditional doctor rather than a health facility or APE? And the other way round?  **Perceptions of fever and its causes**  1. Can you identify a fever? What does a fever usually look like? What tells you that you or a family member has a fever?  2. Do fevers have different causes? What are the main causes of fevers that you know of?  a) If someone talks about malaria, ask about the cause of malaria and the other symptoms of malaria.  3.Do some fevers have to be treated in different ways? If so, give examples. Is it related to the cause of the fever?  a) If someone talks about malaria, ask what they do when they have malaria.  4.Are there fevers that can only be treated by traditional doctors? And only by APEs or in health facility?  **Knowledge of medical care while travelling**  1.Do you always travel to the same places? Where do you travel the most?  2.Do you know people or places that can offer health services and care where you travel?  3.Do you have access to these services? Why? 4.  4.Do you have access to any health services and care while travelling?  5.Are you aware of the health centres on the border? Did you need to use one? Did you use them? Why?  **Attitudes towards fever when travelling**  When you feel you have a fever and are away from home while travelling:  1.Who do you ask for help first (e.g. other travellers, APE, community leader, etc.) and what kind of help do you ask for first (e.g. care at home, medication, diagnosis, etc.)?  2.If you don't initially seek formal/institutional help, what makes you go and seek formal help (e.g. recommendation from a relative, not being able to get out of bed, symptoms other than fever, identifying fever as malaria, etc)?  3.Who is the main person who makes the decision about what to do when you have a fever? At what point?  4.Who is the main person looking after you during an illness while travelling?  5.What is the first form of formal help you seek (e.g. APE, health facility, traditional doctor, community chief, etc)?  a) If not an APE/health facility, what finally makes you go to an APE/health facility? (if anything makes you go to an APE/health facility)  6.How long do you think it takes, in general, from feeling a fever until you make the decision to go to an EPA/health facility? (if you make that decision) And how long does it take from making the decision until you get to an EPA/health facility?  7.Do you have a preference between health centres or APEs for treating fevers? If so, why?  **Barriers and facilitators to seeking care for fevers at the Health Unit or Health Centre**  1.What are the main barriers / difficulties you encounter when you want to seek help at the health facility while travelling when you have a fever? Note: there may be factors related to your personal situation, factors related to the health centre, general factors, others.  2.Do any of these difficulties prevent you from seeking help at a US/CS?  3.What makes it easier for you to seek help at a health facility when you are travelling and have a fever?  **Barriers and facilitators to seeking care for fevers in the APE**  4.What are the main barriers / difficulties you encounter when you want to seek help at the APE when you are travelling and have a fever?  5.Do any of these difficulties prevent you from seeking help from the EPA?  6.What makes it easier for you to seek help from the APE when you are travelling and have a fever?  **Perspectives on the quality and cost of care at US / APEs**  1.When you arrive at the health facility with a fever, what treatment do they give you (explore further, do they do RDT or other tests)?  2.In general, do you think you receive good quality care at the health facility? If yes, why? If not, why not?  3.What about the APE? If yes, why? If not, why not?  4.What are the main costs involved in being cured of a fever if you go to the health facility? What about an APE? Are they affordable for you, or are they a deterrent to seeking health care?  5.Is cost a factor you consider before seeking healthcare? What do you do to cover the cost?  **General recommendations**  1.In your opinion, what needs to be improved to make it easier for the population to access health services? Or is it fine as it is?  2.What needs to be improved for the travelling population to have easy access to health services when they travel and when they arrive at their destination? |
| --- |

**OBSERVATIONS: _____________________________________________________________________________________**

**SIGNATURES**

NAME OF INTERVIEWR: ______________________________ SIGNATURE: _______________________ COD: |__|__|__|__|

S1 Table 3. Semi-structured interview (SSI) guide for teachers

Interviewee data

| **Ref./File/Audio**  **(CARE-SOC-ESE-*NumESE-mmdd*)** | CARE-SOC\|__\|__\|-\|__\|__\|__\|__\| |
| --- | --- |
| **Date** | \|__\|__\|-\|__\|__\|-\|__\|__\|__\|__\| |
| **Specific place** |  |
| **Participant name initials** | \|__\|__\| |
| **Age of the participant** | \|__\|__\|__\| years |
| **Gender of the participant** | □ Male □ Female |
| **Origin** | □ Magude □ Gaza □ Inhambane □ Manhiça □ Moamba □ Maputo city □ Other district of Maputo province □ Rest of Mozambique □ South Africa □ Eswatine □ Other country of Africa □ Other country |
| **How long have you been living in Magude?** | \|__\|__\| months or \|__\|__\|__\| years |
| **Marital status** | □ Single □ Married □Marital Union □ Widower □Other (specify) ______________________ |
| **Number of children under your responsibility** | \|__\|__\|__\| |
| **Level of education** | □ None □ Primary □ Secondary □ Bachelor |
| **Occupation** | □ Housemaid □ Farmer □ Paid farmer □ Student □ Salesperson □ Services  □ Health worker □ Other (specify): ­____________________________________________ |
| **Type of employment** | □ formal work full time □ Formal work partial time □ Informal work (without contract) full time □ Informal work (without contract) partial time □ Sporadic work |
| **Main source of family incomes** | □ Incomes from formal work □ Incomes from informal work □ Agriculture □ Other (specify): ­______________ |
| **How many family members depends on this source of incomes?** | \|__\|__\|__\| |
| **Religion** | □Christian □Muslim □ Hindu □ Animist □ Atheist □ Other (esp): _______________________________ |
| **Posto Administrativo** | □ Magude-sede □ Motaze □ Mahele □ Panjane □ Mapulanguene |
| **Health facility used** | □ Magude-sede □ Chichuco □ Facazissa □ Motaze □ Moine □ Panjane □ Chicutso □ Mahele □ Captine □ Mapulanguene |
| **Distance to health facility** | \|__\|__\|__\| m/km |
| **Type of transport mostly used to health facility** | □ Walking □ Public transport □ Bicycle □ Motorized □ Personal car □ someone’ car □ Never go to health facility |
| **Time to reach to the health facility** | \|__\|__\| min or \|__\|__\| hours |
| **Time to reach community health worker** | \|__\|__\|__\| m/km |
| **Type of transport mostly used to community health worker** | □ Walking □ Public transport □ Bicycle □ Motorized □ Personal car □ Someone’s car □ Never go to community health worker |
| **Time to community health work** | \|__\|__\| min or \|__\|__\| hours |
| **Starting hour of SSI** | \|__\|__\|:\|__\|__\| |
| **Ending hour of SSI** | \|__\|__\|:\|__\|__\| |
| **Result of SSI** | □ Complete □Incomplete, Reasons: ________________________________________________  If applicable, reschedule the date: \|__\|__\|-\|__\|__\|-\|__\|__\|__\|__\| |

Content of interview

**Perceptions about health conditions of concern and the need to seek care in different ways**

1.How do you identify an abnormal or worrying state of health in yourself or a member of your family? What usually indicates to you that you or a family member is unwell?

2.Do you often meet students who are unwell?

3.What are the students' main complaints?

Care provided by the school

4.What does the school do when a student is unwell?

5.Does the school have any form of medical assistance? If so, what is it? (e.g. a medicine kit, a person with first aid knowledge, rapid tests for certain illnesses, etc.)

6.Does the school have any links with health services and care? Do you ever take students to the US/APE? If so, in what cases?

**Perceptions of fever and its causes**

1. Can you identify a fever? What does a fever usually look like? What tells you that you or a family member has a fever?

2.Do fevers have different causes? If so, what are the main causes of fevers that you know of?

a) If talking about malaria, ask about the cause of malaria and the other symptoms of malaria.

3.Are there any fevers that need to be treated in different ways? If so, give examples. Is it related to the cause of the fever?

a) If you're talking about malaria, ask what you do when you have malaria.

4.Are there fevers that can only be treated by traditional doctors? And only by APEs or in health facility?

5. Can you identify when a student in your class is unwell?

a. Can you identify a fever in your students? How? (What tells you it's a fever?)

b. Can you identify malaria in your students? How? (What tells you it's malaria?)

c. If you have malaria, how many school days do you usually miss?

**Attitudes in cases of fever**

6.When a student feels they have a fever, who do they ask for help first and what kind of help do they ask for first?

7.If you don't initially seek help outside the home, what makes you go and seek help outside the home? (e.g. recommendation from the school, recommendation from a relative, not being able to get out of bed, symptoms other than fever, identifying fever as malaria, etc.)

8.Who is the main person who makes the decision about what to do when the student has a fever? At what point?

9.What is the first form of help that students seek outside the home (e.g. APE, health facility, traditional doctor, community leader, etc)?

a) If not an APE/health facility, what finally makes them go to an APE/health facility? (if anything makes them go to an APE/health facility)

10. How long do you think it takes, in general, from the time a student feels a fever until they make the decision to go to an APE/health facility? (if they make that decision) And how long does it take from the time they make the decision until they get to an APE/health facility?

11.Does the school have a preference between health centres or EPAs for treating fevers? If so, why?

**Barriers and facilitators to seeking care for fevers at the Health Unit or Health Centre or APE**

12.What are the main barriers / difficulties you encounter when you want to seek help at the health facility/APE when a student has a fever?

Note: there may be factors related to your personal situation, factors related to the US, general factors, others.

13.Do any of these difficulties prevent you from seeking help at a health facility?

14.What makes it easier for you to seek help at the health facility/APE when a student has a fever?

**General recommendations**

15.In your opinion, what needs to be improved to make it easier for the population to access health services? Or is it good as it is?

**OBSERVATIONS: _____________________________________________________________________________________**

**SIGNATURES**

NAME OF INTERVIEWR: ______________________________ SIGNATURE: _______________________ COD: |__|__|__|__|

S1 Table 4. Semi-structured interview (SSI) guide for health professionals and community health workers

Interviewee data

| **Ref./File/Audio**  **(CARE-SOC-ESE-*NumESE-mmdd*)** | CARE-SOC\|__\|__\|-\|__\|__\|__\|__\| |
| --- | --- |
| **Date** | \|__\|__\|-\|__\|__\|-\|__\|__\|__\|__\| |
| **Specific place** |  |
| **Participant name initials** | \|__\|__\| |
| **Age of the participant** | \|__\|__\|__\| years |
| **Gender of the participant** | □ Male □ Female |
| **Marital status** | □ Single □ Married □Marital Union □ Widower □Other (specify) ______________________ |
| **Level of education** | □ None □ Primary □ Secondary □ Bachelor |
| **Type of health worker** | □ Community health worker (APE) □ Doctor □ Nurse □ Technician □ Other(specify): ­_________ |
| **Type of employment** | □ formal work full time □ Formal work partial time □ Informal work (without contract) full time □ Informal work (without contract) partial time □ Sporadic work |
| **Main source of family incomes** | □ Incomes from formal work □ Incomes from informal work □ Agriculture □ Other (specify): ­______________ |
| **How long have you been working in your actual position** | \|__\|__\| months or \|__\|__\| years |
| **Religion** | □Christian □Muslim □ Hindu □ Animist □ Atheist □ Other (specify): ___________________________ |
| **Starting hour of SSI** | \|__\|__\|:\|__\|__\| |
| **Ending hour of SSI** | \|__\|__\|:\|__\|__\| |
| **Result of SSI** | □ Complete □Incomplete, Reasons: ________________________________________________  If applicable, reschedule the date: \|__\|__\|-\|__\|__\|-\|__\|__\|__\|__\| |

**Content of interview**

**Perceptions of health status and the need to seek care in different ways**

1.What symptoms or illnesses do you frequently attend to?

2.Do you think the population is able to identify fever as an indicator that something is wrong with their health?

3.Apart from having a fever, do people need to have other symptoms to seek help at the health facility or APE? Do people seek help when fever is the only symptom?

4.What are the main causes of fever that you encounter?

5.When the population comes to seek help (at the health facility or APE), after how long with the symptoms do they seek health care? (range)

6.For what kind of illnesses or symptoms do people come quickest? For what kind of illnesses or symptoms do people take the longest to come? (health facility or APE).

7.What are the characteristics of people who most often seek treatment at the beginning of their illness and the characteristics of people who seek treatment when they feel unwell for more than a day?

8.Among the people who come because of fevers and those who come because of other symptoms, which ones seek care quickly?

9.When people come for a fever, do they usually come faster or slower than for other symptoms?

10.Do you think people are able to recognise whether a fever is malaria or another disease?

a) If they suspect it is malaria, does this make people come to health facility/APEs sooner or later? (in terms of time since the onset of illness)

b) If they suspect malaria, is it common for them to seek medication from an informal drug seller instead of seeking care at the health facility/APE?

c) According to your perception, is it more common to seek care at the US/APE or drugs from an informal vendor?

d) What strategy does the Health Unit use to get people to seek health care when they feel they have malaria? What do they recommend?

**Fever routes**

1.When people come for a fever, have they usually visited someone else before? If so, who are they? (e.g. community leader, traditional doctor, grandparent, etc.)

a) Why do you think people visit these people first?

b) In your opinion, do you think these people tell them that they need to seek help at the health facility/APE? Or do you think that these people make it difficult for fever cases to visit the health facility/APE?

2.When you arrive at the health facility/APE, how long do you normally have to wait to be seen? In your opinion, what is the reason for this?

3.When a patient arrives at the health facility or APE with fevers, what treatment do you give them? (Do you do TDR, or do you give them the medicine to treat fevers directly)?

- If you don't do any medical tests, what can you do to make sure that these symptoms aren't malaria?

4.In your opinion, what is the population's reaction to the drug? Do you think they take the full dose? If yes Why / if no Why?

**Barriers and facilitators to seeking care for fevers at the health facility or in an EPA**

1.In your opinion, what barriers does the population encounter in accessing health services and care?

2.In your opinion, what are the circumstances that help people access health services and care?

3.What strategy does the Ministry of Health use to help people access health services and care?

4.Is there anything else that is being done to help people access health services and care (e.g. transport, incentives, food, timetables, etc.)?

5.In your opinion, what is the role of multipurpose agents (APEs) in the community?

- What connection do health professionals have with multipurpose agents? How have they worked?

- Do you think that APEs are important in the community? If yes, why/if no, why not?

- What do you think should be improved about the way APEs work? Or is it fine as it is?

- Do you think that having APEs makes it easier for the community to seek help in cases of fever?

6.Do you think any of these factors affect your decision to seek help at the health facility/APE? If yes, explain why.

- Presence of another woman at home

- Presence of other people at home capable of looking after the family

- Having a stable source of income

- The number of people the family carer has to look after

- Having the support of their husband/wife (financially or practically)

- Distance from health facility / APE

- Transport

- Opening hours

- Technical knowledge or training of health workers

- Waiting time for care

- Tools available at the health facility/APE (diagnosis, medication)

- The way health workers treat people

- How often people at home get sick

- Being familiar with the APE or the Health facility

- Total time the population uses to go to the health facility/APE

**General recommendations**

1.In your opinion, what needs to be improved to make it easier for the population to access health services? Or is it as good as it is?

**OBSERVATIONS:_______________________________________________________________________**

**SIGNATURES**

NAME OF INTERVIEWR: ______________________________ SIGNATURE: _______________________ COD: |__|__|__|__|

S1 Table 5. Semi-structured interview (SSI) guide for traditional healers

Interviewee data

| **Ref./File/Audio**  **(CARE-SOC-ESE-*NumESE-mmdd*)** | CARE-SOC\|__\|__\|-\|__\|__\|__\|__\| |
| --- | --- |
| **Date** | \|__\|__\|-\|__\|__\|-\|__\|__\|__\|__\| |
| **Specific place** |  |
| **Participant name initials** | \|__\|__\| |
| **Age of the participant** | \|__\|__\|__\| years |
| **Gender of the participant** | □ Male □ Female |
| **Marital status** | □ Single □ Married □Marital Union □ Widower □Other (specify) ______________________ |
| **Level of education** | □ None □ Primary □ Secondary □ Bachelor |
| **How long have you been working in your actual position** | \|__\|__\| months or \|__\|__\| years |
| **Religion** | □Christian □Muslim □ Hindu □ Animist □ Atheist □ Other (specify): _______________________________ |
| **Starting hour of SSI** | \|__\|__\|:\|__\|__\| |
| **Ending hour of SSI** | \|__\|__\|:\|__\|__\| |
| **Result of SSI** | □ Complete □Incomplete, Reasons: ________________________________________________  If applicable, reschedule the date: \|__\|__\|-\|__\|__\|-\|__\|__\|__\|__\| |

**Content of interviewer**

1.Is Traditional Healing practice your only job or do you have other jobs?

2.From whom did you learn to be a Traditional Healer?

3.What are the symptoms / conditions / states that you frequently treat?

a) What are the most common causes of these symptoms / conditions / states?

b) What medication do you give for each case?

**Perceptions of fever and its causes**

4. Can you identify a fever? What does a fever usually look like? What tells you that a person has a fever?

5. Do fevers have different causes? What are the main causes of fevers that you know of?

) If you are talking about malaria, ask about the cause of malaria and the other symptoms of malaria.

6.Do some fevers have to be treated in different ways? If so, give examples. Do fevers with different causes have to be treated in different ways?

7. If you're talking about malaria, ask what you do when it's malaria.

8.Are there fevers that can only be treated by traditional doctors? And only by APEs or in health facility? Explain which ones in each case.

**Fever routes**

1. Do many people with a fever visit you?

2.Do you think the population is able to identify fever as an indicator that something is wrong?

3.When people come for a fever, have they usually visited someone else before? If so, who are they? (e.g. community leader, traditional doctor, grandmother, etc.)

4.Why do you think people visit these people first?

5.Do you think that people, in general, visit you before or after going to the US/APE? Why?

6.Do many people with a fever come to see you after you've been to the health facility? If so, why?

7.When people come to you for help because of a fever, after how long with the symptoms do they usually seek help? (range)

8.What are the characteristics of people who most often seek help at the beginning of the illness and the characteristics of people who seek treatment when they feel unwell for another day? Can you describe the differences?

9.Among people who come because of fevers and those who come because of other symptoms, which seek care more quickly?

a) Does it depend on the cause of the fever?

b) Does it depend on the type of person or the person's circumstances?

10. Do you think people are able to recognise whether a fever is malaria or another disease?

a) If they can recognise malaria, does this make people visit Traditional Healers more than health facility/APEs, or vice versa? Why?

b) If you recognise a case of malaria, what do you think is the cause? How do you treat people?

11.Do you sometimes recommend that people look for medicines at the pharmacy or informal drug seller? If so, when?

12.Do you ever recommend that people seek help at health facility or APE? If so, when?

Perspectives on modern health services and care

1.What is your opinion of the medicine practised in Health Units or by APEs?

2.Do you sometimes seek health services and care at health facility or APEs? If so, for what kind of discomfort?

3.Do you have any connection with health services and care?

4.Do you think it is easy or difficult for the population to access health services and care? Why is that?

a) If it is difficult, is it easier to access a traditional doctor? Why is that?

b) Does this make people prefer to visit Traditional Healers?

5.Do you think the population have preference for Traditional Healers or for health facility or APEs?

**Barriers and facilitators to seeking care for fevers at the health facility or in an APE**

1.In your opinion, what barriers does the population encounter in accessing health services and care?

2.In your opinion, what are the circumstances that help people access health services and care?

3.What strategy does the Ministry of Health use to help people access health services and care?

4.Is there anything else that is being done to help people access health services and care? (e.g. transport, incentives, food, timetables, etc.)

**General recommendations**

5.In your opinion, what needs to be improved so that people can access health services easily? Or is it fine as it is?

**OBSERVATIONS:_______________________________________________________________________**

**SIGNATURES**

NAME OF INTERVIEWR: ______________________________ SIGNATURE: _______________________ COD: |__|__|__|__

S1 Table 6. Focus Group discussion for general population

Demographic information of participants

| **Part.** | **Age** | **Sex**  **(F/M)** | **Marital status (*1)** | **Level of education(*2)** | **Occupation**  **(*3)** | **Type of work (*4)** | **Main income of the family (*5)** | **Religion**  **(*6)** | **Posto**  **Administrativo (*7)** |
| --- | --- | --- | --- | --- | --- | --- | --- | --- | --- |
| 1 | \|__\|__\| | \|__\| |  | \|__\| |  |  |  |  | \|__\| |
| 2 | \|__\|__\| | \|__\| |  | \|__\| |  |  |  |  | \|__\| |
| 3 | \|__\|__\| | \|__\| |  | \|__\| |  |  |  |  | \|__\| |
| 4 | \|__\|__\| | \|__\| |  | \|__\| |  |  |  |  | \|__\| |
| 5 | \|__\|__\| | \|__\| |  | \|__\| |  |  |  |  | \|__\| |
| 6 | \|__\|__\| | \|__\| |  | \|__\| |  |  |  |  | \|__\| |
| 7 | \|__\|__\| | \|__\| |  | \|__\| |  |  |  |  | \|__\| |
| 8 | \|__\|__\| | \|__\| |  | \|__\| |  |  |  |  | \|__\| |
| 9 | \|__\|__\| | \|__\| |  | \|__\| |  |  |  |  | \|__\| |
| 10 | \|__\|__\| | \|__\| |  | \|__\| |  |  |  |  | \|__\| |
| 11 | \|__\|__\| | \|__\| |  | \|__\| |  |  |  |  | \|__\| |
| 12 | \|__\|__\| | \|__\| |  | \|__\| |  |  |  |  | \|__\| |
| ***1. Marital status**: 1-Single 2-Married 3-Marital union 4-Widow 5-Other (specify)  ***2. Level of education**: 1-None 2-Primary 3-Secondary 4-Bachelor  ***3. Occupation**: 1- Housemaid 2- Farmer 3- Paid farmer 4- Student 5-Salesperson 6-Services 7-Heaalth worker 8-Other (esp.)  ***4. Type of work:** 1-Formal work full time 2-Fromal work partial time 3-Informal work without contract full time 4-Informal work without contract partial time 5-Sporadic work.  ***5. Main family income:** 1-Formal work 2-Informal work 3-Agriculture 4-Other  ***6. Religion:** 1-Christian 2- Muslim 3-Hindu 4-Animist 5-Atheist 9-Other(specify)  ***7. Posto Administrativo:** 1-Magude Sede 2-Motaze 3-Panjane 4-Mahele 5-Mapulanguene | | | | | | | | | |

DGF data

| **Ref. DGF/File/Audio**  **(CARE-SOC-DGF-*NumDGF-mmdd*)** | CARE-SOC-DGF-\|__\|__\|-\|__\|__\|__\|__\| |
| --- | --- |
| **Date** | \|__\|__\|-\|__\|__\|-\|__\|__\|__\|__\| |
| **Specific local** |  |
| **Initial number of participants** | \|__\|__\| |
| **Final number of participants** | \|__\|__\| |
| **Starting time of DGF** | \|__\|__\|:\|__\|__\| |
| **Ending time of DGF** | \|__\|__\|:\|__\|__\| |
| **Result of DGF** | \|__\| Complete \|__\| Incomplete, reasons:__________________  If applicable, reschedule to: \|__\|__\|-\|__\|__\|-\|__\|__\|__\|__\| |

Content

P**erceptions about health conditions of concern and the need to seek care in different ways**

1. How do you identify an abnormal or worrying state of health in yourself or a member of your family? What usually indicates to you that you or a family member is unwell?

2.Do you think there are any discomforts for which it is necessary to visit a traditional doctor?

3.Do you ever look for medicines directly through an informal drug seller? If so, for what kind of discomfort and why?

4.What kind of discomforts do you think can be treated at home?

5.Have you heard of multi-purpose agents (APEs) that exist in the community? What do you think the role of APEs is in the community? For what kind of discomforts do you go to an APE / take a family member to an APE?

6.For what kind of discomfort do you go to the Health Unit / Health Centre / take a family member to the health facility?

7. If you sometimes visit a traditional doctor, what makes you choose to seek care from a traditional doctor rather than health facility or APE? And the other way round?

**Perceptions of fever and its causes**

1.Can you identify a fever? What does a fever usually look like? What tells you that you or a family member has a fever?

2.What are the main causes of fever that you know of?

a) If someone talks about malaria, ask about the cause of malaria and the other symptoms of malaria.

3.Do some fevers have to be treated in different ways? If so, give examples. Is it related to the cause of the fever?

a) If someone talks about malaria, ask what they do when they have malaria.

b) Are there fevers that can only be treated by traditional doctors? And only by APEs or in health facility? And only in health facility?

**Attitudes towards fever**

**When you or a family member feels you have a fever**:

1.What kind of help do you seek first? (e.g. care at home, medication, diagnosis, talking to the community leader, etc.)

2.What makes you seek help outside the home? (e.g. recommendation from a relative, not being able to get out of bed, symptoms other than fever, identifying fever as malaria, etc)

3.Who is the main person who makes the decision about what to do when someone in the family has a fever? At what point?

4.What is the first form of help you seek outside the home (e.g. APE, health facility, traditional doctor, community leader, etc.)?

a) If not an APE/health facility, what finally makes you go to an APE/health facility? (if anything makes you go to an APE/health facility)

5.How long do you think it takes, in general, from feeling a fever to making the decision to go to an EPA/health facility? (if you make the decision) And how long does it take from making the decision to getting to an APE/health facility?

6.Do you have a preference between health facility(s) and EPAs for the treatment of fevers? If so, why?

**Barriers and facilitators to seeking care for fevers at the Health facility**

1.What are the main barriers/difficulties you encounter when you want to seek help at the health facility for a fever? Note: there may be factors related to your personal situation, factors related to the health facility, general factors, others.

a) Do any of these difficulties prevent you from seeking help at a health facility?

2.What makes it easier for them to seek help at the health facility for fever?

**Barriers and facilitators to seeking care for APE fevers**

3.What are the main barriers/difficulties you encounter when you want to seek help from the APE for fever?

a) Do any of these difficulties prevent you from seeking help from the APE?

4.What makes it easier for you to seek help from the EPA for fever?

5.Do you have family or community support to look after family members when you go to the health facility or APE? If so, from whom do you receive support?

**Perspectives on the quality and cost of care at health facility / APE**

1.When you arrive at the health facility with a fever, what treatment do they give you? (Explore further, do RDTs or other tests?)

2.In general, do you think you receive good quality care at the health facility? If yes, why? If not, why not?

3.What about the APE? If yes, why? If not, why not?

4.What are the main costs involved in being cured of a fever if you go to the health facility? What about an EPA? Are they affordable or high?

5. Is cost a factor you consider before seeking healthcare?

What are the factors that influence your decision to seek help at the health facility /APE?

- Distance from health facility/ APE

- Transport

- Opening hours

- Technical knowledge or training of health workers

- Waiting time

- Tools available at the health facility /APE (diagnosis, medication)

- The way health workers treat people

- How often people at home get sick

- The number of people you have to look after

- Having the support of your husband/wife (financial or practical)

- Being familiar with the APE or the Health facility

- Total time you lose

General recommendations

1.In your opinion, what needs to be improved for the population to have easy access to health services? Or is it fine as it is?

If you didn't identify malaria in the "Perceptions of fever and its causes" section: knowledge of malaria

2.In your opinion, when a person has a fever, chills, headaches and sometimes vomiting, what illness must they have?

3.What else do you know about malaria?

- Causes

- Prevention/ Explore prevention

- Treatment

4.If they didn't use the term malaria, find out if the disease they described is related to malaria or not?

5.In your opinion, what could be the cause and what can be done to eliminate malaria?

6.Generally, when people have the symptoms you mentioned, where do they turn? Include children as well.

- For those who don't go to hospital, what other providers do they go to when they have a fever?

**OBSERVATIONS:_______________________________________________________________________**

**SIGNATURES**

NAME OF INTERVIEWR: ______________________________ SIGNATURE: _______________________ COD: |__|__|_|
