# Supplementary material for: Healthcare-seeking behaviour of fever cases in Magude district, southern Mozambique: A qualitative study
Source: PLoS One. 2025 May 29;20(5):e0323117. doi: 10.1371/journal.pone.0323117 (PMC12122035; doi:10.1371/journal.pone.0323117)
Supplement: S2 Appendix — Semi-structured interviews (SSIs) guides and focus group discussions (FGD) guides used to collect qualitative data, Portuguese version. (DOCX) [file pone.0323117.s002.docx]

**S2 Appendix 2. Data collection tools-Portuguese-version**

S2 Table 1. Guia de entrevistas semi-estruturada (ESE) para população geral (líderes comunitários, chefe do agregado familiar, idosos, mulheres com poder de tomada de decisão, mulheres sem tomada de decisão e mulheres grávidas)

1. **DADOS DA ESE**

| **Ref./Ficheiro/Áudio**  **(CARE-SOC-ESE-*NumESE-mmdd*)** | CARE-SOC\|__\|__\|-\|__\|__\|__\|__\| |
| --- | --- |
| **Data** | \|__\|__\|-\|__\|__\|-\|__\|__\|__\|__\| |
| **Local Específico** |  |
| **Iniciais do nome do participante** | \|__\|__\| |
| **Idade do participante** | \|__\|__\|__\| anos |
| **Género do participante** | □ Masculino □ Feminino |
| **Origem** | □ Magude □ Gaza □ Inhambane □ Manhiça □ Moamba □ Cidade de Maputo □ Outro distrito da Província de Maputo □ Resto de Moçambique □ África do Sul □ Eswatine □ Outro país na Africa □ Outro país no resto do mundo |
| **Há quanto tempo você mora em Magude?** | \|__\|__\| meses ou \|__\|__\|__\| anos |
| **Situação Marital** | □ Solteiro/a □ Casado/a □União □Viúvo/a □Outro (esp) ______________________ |
| **Número de crianças dependentes de si** | \|__\|__\|__\| |
| **Nível de Escolaridade** | □ Nenhum □ Primária □ Secundária □ Superior |
| **Ocupação** | □ Doméstico □ Camponês □ Camponês remunerado □ Estudante □ Negociante □ Serviços  □ Trabalhador da Saúde □ Outro (esp): ­____________________________________________ |
| **Tipo de emprego** | □ Trabalho formal tempo inteiro □ Trabalho formal tempo parcial □ Trabalho informal (sem contrato) tempo inteiro □ Trabalho informal (sem contrato) tempo parcial □ Trabalho pontual ou esporádico |
| **Principal forma de sustento da família** | □ Ingressos do trabalho formal □ Ingressos do trabalho informal □ Agricultura □ Outro (esp): ­______________ |
| **Quantas pessoas dependem dessa forma de sustento?** | \|__\|__\|__\| |
| **Religião** | □Cristão □Islâmica □ Hindu □ Animista □ Ateus □ Outro (esp): _______________________________ |
| **Posto Administrativo** | □ Magude-sede □ Motaze □ Mahele □ Panjane □ Mapulanguene |
| **Unidade Sanitária mais usada** | □ Magude-sede □ Chichuco □ Facazissa □ Motaze □ Moine □ Panjane □ Chicutso □ Mahele □ Captine □ Mapulanguene |
| **Distância a Unidade Sanitária** | \|__\|__\|__\| m/km |
| **Tipo de transporte mais usado para ir a Unidade Sanitária** | □ A pé □ Chapa □ Bicicleta □ Motorizada □ Carro próprio □ Carro de um conhecido □ Nunca vai a US |
| **Tempo que leva para chegar a US** | \|__\|__\| min ou \|__\|__\| horas |
| **Distância ao APE** | \|__\|__\|__\| m/km |
| **Tipo de transporte mais usado para ir ao APE** | □ A pé □ Chapa □ Bicicleta □ Motorizada □ Carro próprio □ Carro de um conhecido □ Nunca vai a US |
| **Tempo que leva para chegar a APE** | \|__\|__\| min ou \|__\|__\| horas |
| **Hora de Início da ESE** | \|__\|__\|:\|__\|__\| |
| **Hora do Fim da ESE** | \|__\|__\|:\|__\|__\| |
| **Resultado da ESE** | □ Completa □Incompleta, razões: ________________________________________________  Se aplicável, remarcada para: \|__\|__\|-\|__\|__\|-\|__\|__\|__\|__\| |

1. **CONTEÚDOS DA ENTREVISTA**

| **Percepções sobre estados de saúde preocupantes e necessidade de procura de cuidados em diferentes formas**   1. Como você identifica um estado de saúde anormal ou preocupante em si ou em um membro da sua família? O que lhe indica, normalmente, que você ou um membro da família não está bem? 2. Como descreveria o seu estado de saúde geral? (bom, mau, normal) 3. Acha que há alguns desconfortos para os quais é preciso visitar um médico tradicional? Esses desconfortos podem ser resolvidos também numa Unidade Sanitária (US) ou por um APE? 4. Para que tipo de desconfortos você procura medicamentos directamente a través dum vendedor informal de fármacos? Porque? 5. Que tipo de desconfortos acha que pode-se tratar em casa? 6. Já ouviu falar de agentes polivalentes (APEs) que existem na comunidade? Para que tipo de desconfortos você vai a um APE? Para que tipo de desconfortos você leva a uma criança a um APE? 7. Para que tipo de desconfortos você vai a Unidade Sanitária / Centro de Saúde? Para que tipo de desconfortos você leva a uma criança a US/CS? 8. Se as vezes visita um médico tradicional, o que lhe faz escolher entre procurar cuidados num médico tradicional e não numa US/CS ou APE? E ao revés? Contribuissemos   **Percepções sobre a febre e as suas causas**   1. Você pode identificar uma febre? Como se apresenta a febre, normalmente? O que lhe indica que você ou um membro da família tem febre? 2. Quais são as principais causas de febres que você conhece?    1. *Se alguém fala de malária, perguntar sobre a causa da malária e os outros sintomas da malária.* 3. Há febres que têm de ser atendidas de diferentes modos? Se sim, dê exemplos. Está relacionado com a causa da febre?    1. *Se alguém fala de malária, perguntar o que faz quando tem malária.*    2. *Há febres que só podem ser atendidas pelos médicos tradicionais? E só por APEs ou em US/CS? E só em US/CS?*   **Atitudes em casos de febre**  Quando você sente que têm febre, ou um membro da família sente que têm febre:   1. A quem pede ajuda primeiro (ex. outro membro da família, APE, chefe comunitário, etc) e que tipo de ajuda procura inicialmente (ex. cuidados em casa, medicamentos, diagnostico, etc)? 2. O que lhe leva a procurar ajuda fora de casa? (ex. recomendação dum familiar, não poder sair da cama, outros sintomas além da febre, identificar a febre como malária, etc) 3. Quem é a principal pessoa que toma a decisão sobre o que fazer quando você tem febre? E quando uma criança tem febre? Em que momento? 4. Quem é a principal pessoa que cuida dos doentes em casa? 5. Qual é a primeira forma de ajuda que você procura fora de casa (ex. APE, US/CS, medico tradicional, chefe comunitário, etc)?    1. Se não for APE/US/CS, o que lhe faz finalmente ir a um APE/US/CS? (se alguma coisa lhe faz ir a APE/US/CS) 6. Quanto tempo acha que demora, em geral, desde que sente uma febre até se tomar a decisão de ir a um APE/US? (se toma essa decisão) E quanto tempo demora desde que se toma a decisão até chegar a um APE/US? 7. Têm alguma preferência entre Unidade Sanitária(s) vs APEs para o tratamento das febres? Se sim, a que se deve? 8. Se tem um familiar que foi diagnosticado malária, a sua volta para casa, encontra outro membro de família com febres, qual seria a sua reação?   **Barreiras e facilitadores da procura de cuidados de febres na Unidade Sanitária ou Centro de Saúde**   1. Quais são as principais barreiras / dificuldades que você encontra quando quere procurar ajuda na US/CS quando você ou um membro da família tem febre? *Nota: pode haver fatores da sua situação pessoal, fatores relacionados com a US, fatores gerais, outros*.    1. *Algumas destas dificuldades lhe impedem procurar ajuda numa US/CS?* 2. O que lhe facilita a procura de ajuda na US/CS quando você ou um membro da família tem febre?   **Barreiras e facilitadores da procura de cuidados de febres do APE**   1. Quais são as principais barreiras / dificuldades que você encontra quando quere procurar ajuda do APE quando você ou um membro da família tem febre?    1. *Algumas destas dificuldades lhe impedem procurar ajuda do APE?* 2. O que lhe facilita a procura de ajuda do APE quando você ou um membro da família tem febre? 3. Tem apoio na família ou na comunidade para cuidar do resto de membros da família se você vai a US ou APE? Se sim, de quem recebe apoio? (Se o entrevistado for mulher: o marido está em casa normalmente?)   **Perspectivas sobre a qualidade e custo do atendimento nas US / APEs**   1. Quando chega na US/CS, com febres, qual é o tratamento que vos dão? (Explore mais, se fazem TDR, ou outros exames?) 2. No geral, você considera que recebe um atendimento de boa qualidade na US/CS? Se sim, porque? Se não, porque não? 3. E do APE? Se sim, porque? Se não, porque não? 4. Quais são os principais custos envolvidos no processo de se curar duma febre se vai a US/CS? E a um APE? São acessíveis para si, ou são um impedimento para procurar cuidados de saúde? 5. O custo é um fator que você considera antes de procurar cuidados de saúde? O que faz para cobrir o custo?   Quais são os factores que influenciam na tomada de decisao para procurar ajuda na US/APE?   - Distância da US/CS / APE - Transporte - Horário de atendimento - Conhecimentos técnicos ou formação dos trabalhadores de saúde - Tempo de espera ao atendimento - Ferramentas disponíveis na US/APE (diagnostico, medicamento) - A maneira de tratar as pessoas que os trabalhadores de saúde têm - A frequência com que pessoas em casa ficam doentes - O número de pessoas que você há de cuidar - Ter o apoio do seu marido/mulher (econômico ou a nível prático) - Estar familiarizado com o APE ou a Unidade Sanitária/CS - Tempo total que você perde   **Recomendações gerais**   1. Na sua opinião, o que tem que se melhorar para a população ter acesso aos serviços de saúde com facilidade? Ou está bom como está?   **Caso não tenham identificado a malária na parte de “Percepções sobre a febre e suas causas”: conhecimentos sobre a malária**   1. Na sua opinião, quando uma pessoa tem febres, arrepios de frio, dores de cabeça e as vezes vómitos, que doença deve ter? 2. O que sabe mais sobre a malária? 3. Caso refira outra doença, explore mais, pergunte se essa doença tem haver com a malária?   • Causa  • Prevenção/ Explorar mais sobre a prevenção  • Tratamento   1. Na sua opinião, qual pode ser a causa e o que pode-se fazer para eliminar a malária? |
| --- |

1. **OBSERVAÇÕES ______________________________________________________________**

**ASSINATURAS**

NOME DO ENTREVISTADOR: __________________________ Assinatura: _______________________ CÓDIGO: |__|__|__|__|

S2 Table 2. Guia de entrevistas semi-estruturada (ESE) para viajantes

Dados da ESSE

| **Ref./Ficheiro/Áudio**  **(CARE-SOC-ESE-*NumESE-mmdd*)** | CARE-SOC-ESE-\|__\|__\|-\|__\|__\|__\|__\| |
| --- | --- |
| **Data** | \|__\|__\|-\|__\|__\|-\|__\|__\|__\|__\| |
| **Local Específico** |  |
| **Iniciais do nome do participante** | \|__\|__\| |
| **Idade do participante** | \|__\|__\|__\| anos |
| **Género do participante** | □ Masculino □ Feminino |
| **Origem** | □ Magude □ Gaza □ Inhambane □ Manhiça □ Moamba □ Cidade de Maputo □ Outro distrito da Província de Maputo □ Resto de Moçambique □ África do Sul □ Eswatine □ Outro país na África □ Outro país no resto do mundo |
| **Há quanto tempo você mora em Magude?** | \|__\|__\| meses ou \|__\|__\|__\| anos |
| **Situação Marital** | □ Solteiro/a □ Casado/a □União □Viúvo/a □Outro (esp) ______________________ |
| **Número de crianças dependentes de si** | \|__\|__\|__\| |
| **Nível de Escolaridade** | □ Nenhum □ Primária □ Secundária □ Superior |
| **Ocupação** | □ Doméstico □ Camponês □ Camponês remunerado □ Estudante □ Negociante □ Serviços  □ Trabalhador da Saúde □ Outro (esp): ­____________________________________________ |
| **Tipo de emprego** | □ Trabalho formal tempo inteiro □ Trabalho formal tempo parcial □ Trabalho informal (sem contrato) tempo inteiro □ Trabalho informal (sem contrato) tempo parcial □ Trabalho pontual ou esporádico |
| **Principal forma de sustento da família** | □ Ingressos do trabalho formal □ Ingressos do trabalho informal □ Agricultura □ Outro (esp): ­______________ |
| **Quantas pessoas dependem dessa forma de sustento?** | \|__\|__\|__\| |
| **Religião** | □Cristão □Islâmica □ Hindu □ Animista □ Ateus □ Outro(esp): _______________________________ |
| **Posto Administrativo** | □ Magude-sede □ Motaze □ Mahele □ Panjane □ Mapulanguene |
| **Unidade Sanitária mais usada** | □ Magude-sede □ Chichuco □ Facazissa □ Motaze □ Moine □ Panjane □ Chicutso □ Mahele □ Captine □ Mapulanguene |
| **Distância a Unidade Sanitária** | \|__\|__\|__\| m ou \|__\|__\|__\| km |
| **Meios mais usado para ir a Unidade Sanitária** | □ A pé □ Chapa □ Bicicleta □ Motorizada □ Carro próprio □ Carro de um conhecido □ Nunca vai a US |
| **Tempo que leva para chegar a US** | \|__\|__\| min ou \|__\|__\| horas |
| **Distância ao APE** | \|__\|__\|__\| m ou km \|__\|__\|__\| □N/A |
| **Meios mais usado para ir ao APE** | □ A pé □ Chapa □ Bicicleta □ Motorizada □ Carro próprio □ Carro de um conhecido □ Nunca vai a US □ N/A |
| **Tempo que leva para chegar a APE** | \|__\|__\| min ou \|__\|__\| horas □N/A |
| **Hora de Início da ESE** | \|__\|__\|:\|__\|__\| |
| **Hora do Fim da ESE** | \|__\|__\|:\|__\|__\| |
| **Resultado da ESE** | □ Completa □Incompleta, razões: ________________________________________________  Se aplicável, remarcada para: \|__\|__\|-\|__\|__\|-\|__\|__\|__\|__\| |

1. **CONTEÚDOS DA ENTREVISTA**

| **Percepções sobre estados de saúde preocupantes e necessidade de procura de cuidados em diferentes formas**   1. Como você identifica um estado de saúde anormal ou preocupante em si ou em um membro da sua família? O que lhe indica, normalmente, que você não está bem? 2. Como descreveria o seu estado de saúde geral? (bom, mau, normal) 3. Para que tipo de desconfortos você vai a um médico tradicional? Para que tipo de desconfortos você leva a uma criança um médico tradicional? Esses desconfortos podem ser resolvidos também numa Unidade Sanitária (US) ou por um APE? 4. Para que tipo de desconfortos você procura medicamentos diretamente a través dum vendedor de medicamentos? Porque? 5. Para que tipo de desconfortos você fica com cuidados em casa? 6. Já ouviu falar de agentes polivalentes (APEs) que existem na comunidade? Para que tipo de desconfortos você vai a um APE? Para que tipo de desconfortos você leva a uma criança a um APE? 7. Para que tipo de desconfortos você vai a Unidade Sanitária / Centro de Saúde? Para que tipo de desconfortos você leva a uma criança a US/CS? 8. O que lhe faz escolher entre procurar cuidados num médico tradicional e não numa US/CS ou APE? E ao inverso?   **Percepções sobre a febre e as suas causas**   1. Você pode identificar uma febre? Como se apresenta a febre, normalmente? O que lhe indica que você ou um membro da família tem febre? 2. Há febres que têm diferentes causas? Quais são as principais causas de febres que você conhece?    1. *Se alguém fala de malária, perguntar sobre a causa da malária e os outros sintomas da malária.* 3. Há febres que têm de ser atendidas de diferentes modos? Se sim, dê exemplos. Está relacionado com a causa da febre?    1. *Se alguém fala de malária, perguntar o que faz quando tem malária.* 4. Há febres que só podem ser atendidas pelos médicos tradicionais? E só por APEs ou em US/CS?   **Conhecimentos dos atendimentos médicos durante as viagens**   1. Costuma viajar sempre para mesmos lugares? Aonde é que mais viaja? 2. Conhece pessoas ou lugares que podem oferecer serviços e cuidados de saúde lá onde você viaja? 3. Você tem aceso a esses serviços? Porque? 4. Tem aceso a algum serviço e cuidado de saúde durante a viagem? 5. Tem conhecimento sobre os postos de saúde da fronteira? Teve necessidade de usar algum? Usou-o? Porque?   **Atitudes em casos de febre na última viagem**  Quando você sente que têm febre, e está fora de casa durante uma viagem:   1. A quem pede ajuda primeiro (ex. outros viajantes, APE, chefe comunitário, etc) e que tipo de ajuda pede inicialmente (ex. cuidados em casa, medicamentos, diagnostico, etc)? 2. Se inicialmente não procura ajuda formal/institucional, o que lhe faz ir procurar ajuda formal? (ex. recomendação dum familiar, não poder sair da cama, outros sintomas além da febre, identificar a febre como malária, etc) 3. Quem é a principal pessoa que toma a decisão sobre o que fazer quando você tem febre? Em que momento? 4. Quem é a principal pessoa que cuida de si durante uma doença na viagem? 5. Qual é a primeira forma de ajuda formal que você procura (ex. APE, US/CS, medico tradicional, chefe comunitário, etc)?    1. Se não for APE/US/CS, o que lhe faz finalmente ir a um APE/US/CS? (se alguma coisa lhe faz ir a APE/US/CS) 6. Quanto tempo acha que demora, em geral, desde que sente uma febre até se tomar a decisão de ir a um APE/US? (se toma essa decisão) E quanto tempo demora desde que se toma a decisão até chegar a um APE/US? 7. Têm alguma preferência entre Unidade Sanitária(s) ou APEs para o tratamento das febres? Se sim, a que se deve?   **Barreiras e facilitadores da procura de cuidados de febres na Unidade Sanitária ou Centro de Saúde**   1. Quais são as principais barreiras / dificuldades que você encontra quando quer procurar ajuda na US/CS durante a viajando quando tem febre? *Nota: pode haver fatores da sua situação pessoal, fatores relacionados com a US, fatores gerais, outros*. 2. Algumas destas dificuldades lhe impedem procurar ajuda numa US/CS? 3. O que lhe facilita a procura de ajuda na US/CS quando você está viajando e tem febre?   **Barreiras e facilitadores da procura de cuidados de febres no APE**   1. Quais são as principais barreiras / dificuldades que você encontra quando quer procurar ajuda no APE quando você está viajando e tem febre? 2. Algumas destas dificuldades lhe impedem procurar ajuda do APE? 3. O que lhe facilita a procura de ajuda do APE quando você está viajando e tem febre?   **Perspectivas sobre a qualidade e custo do atendimento nas US / APEs**   1. Quando chega na US/CS, com febres, qual é o tratamento que lhe dão? (Explore mais, se fazem TDR, ou outros exames?) 2. No geral, você considera que recebe um atendimento de boa qualidade na US/CS? Se sim, porque? Se não, porque não? 3. E do APE? Se sim, porque? Se não, porque não? 4. Quais são os principais custos envolvidos no processo de se curar duma febre se vai a US/CS? E a um APE? São acessíveis para si, ou são um impedimento para procurar cuidados de saúde? 5. O custo é um fator que você considera antes de procurar cuidados de saúde? O que faz para cobrir o custo?   **Recomendações gerais**   1. Na sua opinião, o que tem se melhorar para a população ter acesso aos serviços de saúde com facilidade? Ou está bom como está? 2. O que tem que se melhorar para a população que viaja ter acesso aos serviços de saúde com facilidade quando viaja e quando chega ao destino da viagem? |
| --- |

**OBSERVAÇÕES ________________________________________________________________________________**

**ASSINATURAS**

NOME DO ENTREVISTADOR: ______________________________ Assinatura: _______________________ CÓDIGO: |__|__|__|__|

S2 Table 3. Guia de entrevistas semi-estruturada (ESE) para professores

Dados da ESSE

| **Ref./Ficheiro/Áudio**  **(CARE-SOC-ESE-*NumESE-mmdd*)** | CARE-SOC-ESE-\|__\|__\|-\|__\|__\|__\|__\| |
| --- | --- |
| **Data** | \|__\|__\|-\|__\|__\|-\|__\|__\|__\|__\| |
| **Local Específico** |  |
| **Iniciais do nome do participante** | \|__\|__\| |
| **Idade do participante** | \|__\|__\|__\| anos |
| **Género do participante** | □ Masculino □ Feminino |
| **Origem** | □ Magude □ Gaza □ Inhambane □ Manhiça □ Moamba □ Cidade de Maputo □ Outro distrito da Província de Maputo □ Resto de Moçambique □ África do Sul □ Eswatine □ Outros país na África □ Outro país no resto do mundo |
| **Há quanto tempo você mora em Magude?** | \|__\|__\| meses ou \|__\|__\|__\| anos |
| **Situação Marital** | □ Solteiro/a □ Casado/a □União □Viúvo/a □Outro (esp) ______________________ |
| **Número de crianças dependentes de si** | \|__\|__\|__\| |
| **Nível de Escolaridade** | □ Nenhum □ Primária □ Secundária □ Superior |
| **Ocupação** | □ Doméstico □ Camponês □ Camponês remunerado □ Estudante □ Negociante □ Serviços  □ Trabalhador da Saúde □ Outro (esp): ­____________________________________________ |
| **Tipo de emprego** | □ Trabalho formal tempo inteiro □ Trabalho formal tempo parcial □ Trabalho informal (sem contrato) tempo inteiro □ Trabalho informal (sem contrato) tempo parcial □ Trabalho pontual ou esporádico |
| **Principal forma de sustento da família** | □ Ingressos do trabalho formal □ Ingressos do trabalho informal □ Agricultura □ Outro (esp): ­______________ |
| **Quantas pessoas dependem dessa forma de sustento ou fonte de ingressos?** | \|__\|__\|__\| |
| **Religião** | □Cristão □Islâmica □ Hindu □ Animista □ Ateus □ Outro(esp): _______________________________ |
| **Posto Administrativo** | □ Magude-sede □ Motaze □ Mahele □ Panjane □ Mapulanguene |
| **Unidade Sanitária mais usada** | □ Magude-sede □ Chichuco □ Facazissa □ Motaze □ Moine □ Panjane □ Chicutso □ Mahele □ Captine □ Mapulanguene |
| **Distância a Unidade Sanitária** | \|__\|__\|__\| km |
| **Meios mais usado para ir a Unidade Sanitária** | □ A pé □ Chapa □ Bicicleta □ Motorizada □ Carro próprio □ Carro de um conhecido □ Nunca vai a US |
| **Tempo que leva para chegar a US** | \|__\|__\| min ou \|__\|__\| horas |
| **Distância ao APE** | \|__\|__\|__\|m ou \|__\|__\|__\| km □N/A |
| **Meios mais usado para ir ao APE** | □ A pé □ Chapa □ Bicicleta □ Motorizada □ Carro próprio □ Carro de um conhecido □ Nunca vai a US |
| **Tempo que leva para chegar a APE** | \|__\|__\| min ou \|__\|__\| horas |
| **Hora de Início da ESE** | \|__\|__\|:\|__\|__\| |
| **Hora do Fim da ESE** | \|__\|__\|:\|__\|__\| |
| **Resultado da ESE** | □ Completa □Incompleta, razões: ________________________________________________  Se aplicável, remarcada para: \|__\|__\|-\|__\|__\|-\|__\|__\|__\|__\| |

1. **CONTEÚDOS DA ENTREVISTA**

| **Percepções sobre estados de saúde preocupantes e necessidade de procura de cuidados em diferentes formas**   1. Como você identifica um estado de saúde anormal ou preocupante em si ou em um membro da sua família? O que lhe indica, normalmente, que você ou um membro da família não está bem? 2. É frequente encontrar alunos que não se encontram bem de saúde? 3. Quais são as principais queixas dos estudantes?   **Atendimentos prestados pela escola**   1. O que é que a escola faz quando um aluno não se encontra bem de saúde? 2. A escola tem alguma forma de atendimento médico? Se sim, qual? (ex. kit de remédios, uma pessoa que tem conhecimentos de primeiros auxílios, testes rápidos para algumas doenças, etc) 3. A escola tem alguma ligação com os serviços e cuidados de saúde? Alguma vez levam alunos a US/APE? Se sim, em que casos?   **Percepções sobre a febre e as suas causas**   1. Você pode identificar uma febre? Como se apresenta a febre, normalmente? O que lhe indica que você ou um membro da família tem febre? 2. Há febres que têm diferentes causas? Se sim, quais são as principais causas de febres que você conhece?    1. *Se fala de malária, perguntar sobre a causa da malária e os outros sintomas da malária.* 3. Há febres que têm de ser atendidas de diferentes modos? Se sim, dê exemplos. Está relacionado com a causa da febre?    1. *Se fala de malária, perguntar o que faz quando tem malária.* 4. Há febres que só podem ser atendidas pelos médicos tradicionais? E só por APEs ou em US/CS? 5. Você consegue identificar quando um aluno na sua aula não está bem de saude?    1. Consegue identificar uma febre nos seus alunos? Como? (o que lhe indica que é uma febre?)    2. Consegue identificar uma malária nos seus alunos? Como? (o que lhe indica que é uma malária?)    3. Se tem malária, quantos dias de escola costuma a perder?   **Atitudes em casos de febre**   1. Quando um aluno sente que têm febre, a quem pede ajuda primeiro e que tipo de ajuda pede inicialmente? 2. Se inicialmente não procura ajuda fora de casa, o que lhe faz ir procurar ajuda fora de casa? (ex. recomendação da escola, recomendação dum familiar, não poder sair da cama, outros sintomas além da febre, identificar a febre como malária, etc) 3. Quem é a principal pessoa que toma a decisão sobre o que fazer quando o aluno tem febre? Em que momento? 4. Qual é a primeira forma de ajuda que os alunos procuram fora de casa (ex. APE, US/CS, medico tradicional, líder comunitário, etc)?    1. Se não for APE/US/CS, o que lhes faz finalmente ir a um APE/US/CS? (se alguma coisa lhe faz ir a APE/US/CS) 5. Quanto tempo acha que demora, em geral, desde que o aluno sente uma febre até se tomar a decisão de ir a um APE/US? (se toma essa decisão) E quanto tempo demora desde que se toma a decisão até chegar a um APE/US? 6. A escola têm alguma preferência entre Unidade Sanitária(s) ou APEs para o tratamento das febres? Se sim, a que se deve?   **Barreiras e facilitadores da procura de cuidados de febres na Unidade Sanitária ou Centro de Saúde ou APE**   1. Quais são as principais barreiras / dificuldades encontradas quando quer procurar ajuda na US/CS/APE quando um aluno tem febre?   *Nota: pode haver fatores da sua situação pessoal, fatores relacionados com a US, fatores gerais, outros*.   1. Algumas destas dificuldades lhe impedem procurar ajuda numa US/CS? 2. O que lhe facilita a procura de ajuda na US/CS/APE quando um aluno tem febre?   **Recomendações gerais**   1. Na sua opinião, o que tem que se melhorar para que a população tenha acesso aos serviços de saúde com facilidade? Ou está bom como está? |
| --- |

**OBSERVAÇÕES**

**ASSINATURAS**

NOME DO ENTREVISTADOR: ______________________________ Assinatura: _______________________ CÓDIGO: |__|__|__|__|

S2 Table 4. Guia de entrevistas semi-estruturada (ESE) para profissionais de saúde

Dados da ESSE

| **Ref./Ficheiro/Áudio**  **(CARE-SOC-ESE-*NumESE-mmdd*)** | CARE-SOC-ESE-\|__\|__\|-\|__\|__\|__\|__\| |
| --- | --- |
| **Data** | \|__\|__\|-\|__\|__\|-\|__\|__\|__\|__\| |
| **Local Específico** |  |
| **Iniciais do nome do participante** | \|__\|__\| |
| **Género do participante** | □ Masculino □ Feminino |
| **Situação Marital** | □ Solteiro/a □ Casado/a □União □Viúvo/a □Outro (esp) ______________________ |
| **Nível de Escolaridade** | □ Nenhum □ Primária □ Secundária □ Superior |
| **Tipo de trabalhador de saúde** | □ Agente Polivalente Elementar (APE) □ Medico/a □ Enfermeiro/a □ Técnico/a de medicina □ Outro (esp): ­____________________________________________ |
| **Há quanto tempo está no seu actual posto?** | \|__\|__\| meses ou \|__\|__\| anos |
| **Religião** | □Cristão □Islâmica □ Hindu □ Animista □ Ateus □ Outro(esp): _________ |
| **Hora de Início da ESE** | \|__\|__\|:\|__\|__\| |
| **Hora do Fim da ESE** | \|__\|__\|:\|__\|__\| |
| **Resultado da ESE** | □ Completa □Incompleta, razões: ________________________________________________  Se aplicável, remarcada para: \|__\|__\|-\|__\|__\|-\|__\|__\|__\|__\| |

1. **CONTEÚDOS DA ENTREVISTA**

| **Percepções sobre estados de saúde e necessidade de procura de cuidados em diferentes formas**   1. Quais são os sintomas ou doenças que você atende frequentemente? 2. Acha que a população é capaz de identificar a febre como um indicador que alguma coisa não está bem na sua saúde? 3. Para além de ter febre, a população precisa de ter outros sintomas para procurar ajuda na US ou APE? Existem pessoas que procuram ajuda quando a febre é o único sintoma? 4. Quais são as principais causas de febre que você encontra? 5. Quando a população vem procurar ajuda (na US ou APE), Depois de quanto tempo com os sintomas é que procura cuidados de saúde? (intervalo) 6. Para que tipo de doenças ou sintomas as pessoas vem mais rápido? Para que tipo de doenças ou sintomas as pessoas demoram mais em vir? (US ou APE). 7. Quais são as características das pessoas que mais procuram o tratamento no início da doença e características das pessoas que procuram o tratamento quando sentem-se mal por mais de dia? 8. Entre as pessoas que vem por causa de febres e os que vem por causa de outros sintomas, quais são os que procuram cuidados rapidamente? 9. Quando as pessoas vêm por causa de uma febre, costumam a vir mais rápido ou mais lento do que por outros sintomas? 10. Acha que as pessoas são capazes de reconhecer se uma febre é malária ou outra doença?     1. Se suspeitam que seja a malária, isto faz com que as pessoas venham mais cedo ou mais tarde a US/APEs? (em termos de tempo desde o início da doença)     2. Se suspeitam malária, é comum que procurem medicamentos num vendedor informal de fármacos ao em vez de procurar atendimento na US/APE?     3. Segundo sua percepção, é mais comum procurar atendimento na US/APE ou fármacos num vendedor informal?     4. Qual é a estratégia usada pela Unidade Sanitária para que as pessoas procurem cuidados de saúde quando sentem que têm malária? O que recomendam?   **Itinerários das febres**   1. Quando as pessoas vêm por causa de uma febre, costumam ter visitado alguma outra pessoa antes? Se sim, quem são essas pessoas? (ex. chefe comunitário, médico tradicional, avo, etc)    1. Por que é que pensa que a população visita primeiro essas pessoas?    2. Na sua opinião, acha que essas pessoas lhes indicam que têm de procurar ajuda na US/APE? Ou consideram que essas pessoas dificultam que os casos de febre visitem a US/APE? 2. Ao chegar a US/APE, quanto tempo têm de esperar a ser atendidos normalmente? Na sua opinião, a que se deve esse tempo? 3. Quando um paciente chega na US/CS ou APE com febres, qual é o tratamento que dão? (Se faz TDR, ou dão o medicamento para tratar febres directamente)?  - Se não faz nenhum exame médico, explore o que lhe garante que esses sintomas não se tratam de malária?  1. Na sua opinião, qual é a reação da população ao medicamento? Acha que tomam a dose completa? Se sim Porquê / se não porquê?   **Barreiras e facilitadores da procura de cuidados de febres na US/CS ou num APE**   1. Na sua opinião, quais são as barreiras que a população encontra para ter acesso aos serviços e cuidados de saúde? 2. Na sua opinião, quais são as circunstâncias que ajudam as pessoas a ter acesso aos serviços e cuidados de saúde? 3. Qual é a estratégia usada pelo Ministério de Saúde para que as pessoas tenham acesso aos serviços e cuidados de saúde? 4. Há outra coisa que está a ser feita para que as pessoas tenham acesso aos serviços e cuidados de saúde? (ex. transporte, incentivos, comida, horários, etc) 5. Na sua opinião, qual é o papel dos agentes polivalentes (APEs) na comunidade?  - Qual é a ligação que os profissionais de saúde têm com agentes polivalentes? Como é que tem trabalhado? - Acha que os APEs, são importantes na comunidade? Se sim Porquê/ se não porquê? - O que acha que devia-se melhorar em relação a maneira de trabalhar dos APEs? Ou está bem como está? - Acha que ter APEs facilita a comunidade procurar ajuda em casos de febre?  1. Acha que algum destes factores afecta na decisão de procurar ajuda na US/APE? Se sim, explique porque.  - Presença de outra mulher em casa - Presença de outras pessoas em casa capazes de cuidar da família - Ter uma fonte de ingressos estável - O número de pessoas que a pessoa que cuida a família há de cuidar - Ter o apoio do seu marido/mulher (econômico ou a nível prático) - Distância da US/CS / APE - Transporte - Horário de atendimento - Conhecimentos técnicos ou formação dos trabalhadores de saúde - Tempo de espera ao atendimento - Ferramentas disponíveis na US/APE (diagnostico, medicamento) - A maneira de tratar as pessoas que os trabalhadores de saúde têm - A frequência com que pessoas em casa ficam doentes - Estar familiarizado com o APE ou a Unidade Sanitária/CS - Tempo total que a população usa para ir a US/APE   **Recomendações gerais**   1. Na sua opinião, o que tem se melhorar para a população ter acesso aos serviços de saúde com facilidade? Ou está bom como está? |
| --- |

**OBSERVAÇÕES __________________________________________________________**

1. **ASSINATURAS**

NOME DO ENTREVISTADOR: _____________________ Assinatura: _______________________ CÓDIGO: |__|__|__|__|

S2 Table 5. Guia de entrevistas semi-estruturada (ESE) para médicos tradicionais

Dados aa ESE

| **Ref./Ficheiro/Áudio**  **(CARE-SOC-ESE-*NumESE-mmdd*)** | CARE-SOC-ESE-\|__\|__\|-\|__\|__\|__\|__\| |
| --- | --- |
| **Data** | \|__\|__\|-\|__\|__\|-\|__\|__\|__\|__\| |
| **Local Específico** |  |
| **Posto administrativo** | □ Magude sede □ Mahele □ Panjane □ Motaze □ Mapulanguene |
| **Iniciais do nome do participante** | \|__\|__\| |
| **Género do participante** | □ Masculino □ Feminino |
| **Situação Marital** | □ Solteiro/a □ Casado/a □União □Viúvo/a □Outro (esp) ______________________ |
| **Nível de Escolaridade** | □ Nenhum □ Primária □ Secundária □ Superior |
| **Há quanto tempo está a fazer o seu trabalho de Médico Tradicional em Magude?** | \|__\|__\| meses ou \|__\|__\| anos |
| **Religião** | □Cristão □Islâmica □ Hindu □ Animista □ Ateus □ Outro(esp): |
| **Hora de Início da ESE** | \|__\|__\|:\|__\|__\| |
| **Hora do Fim da ESE** | \|__\|__\|:\|__\|__\| |
| **Resultado da ESE** | □ Completa □Incompleta, razões: ___________________________________________  Se aplicável, remarcada para: \|__\|__\|-\|__\|__\|-\|__\|__\|__\|__\| |

2. Conteúdo da entrevista

| **Introdução**   1. Ser Médico Tradicional é o seu único emprego ou tem outros empregos? 2. De quem aprendeu para ser Médico Tradicional? 3. Quais são os sintomas / condições / estados que você atende frequentemente?    1. Quais são as causas mais comuns desses sintomas / condições / estados?    2. Qual o medicamento que você dá para cada caso?   **Percepções sobre a febre e as suas causas**   1. Você pode identificar uma febre? Como se apresenta a febre, normalmente? O que lhe indica que uma pessoa tem febre? 2. Há febres que têm diferentes causas? Quais são as principais causas de febres que você conhece?    1. *Se fala de malária, perguntar sobre a causa da malária e os outros sintomas da malária.* 3. Há febres que têm de ser atendidas de diferentes modos? Se sim, dê exemplos. Febres com diferentes causas têm de ser atendidas de diferentes modos? 4. *Se fala de malária, perguntar o que faz quando é malária.* 5. Há febres que só podem ser atendidas pelos médicos tradicionais? E só por APEs ou em US/CS? Explique quais em cada caso.   **Itinerários das febres**   1. Há muita gente com febre que lhe visita? 2. Acha que a população é capaz de identificar a febre como um indicador que alguma coisa não está bem? 3. Quando as pessoas vêm por causa de uma febre, costumam ter visitado alguma outra pessoa antes? Se sim, quem são essas pessoas? (ex. líder comunitário, médico tradicional, avó, etc) 4. Porquê acha que a população visita primeiro essas pessoas? 5. Acha que as pessoas , no geral, lhe visita antes ou depois de ir a US/APE? Porque? 6. Há muitas pessoas com febre que vem lhe visitar depois de ter ido a US/APE? Se sim, porquê? 7. Quando a população vem procurar ajuda por causa da febre, depois de quanto tempo com os sintomas é que procuram a sua ajuda normalmente? (intervalo) 8. Quais são as características das pessoas que mais procuram ajuda no início da doença e características das pessoas que procuram o tratamento quando sentem-se mal por mais um dia? Descreve as diferenças? 9. Entre as pessoas que vem por causa de febres e os que vem por causa de outros sintomas, quais procuram cuidados mais rapidamente?    1. Depende da causa da febre?    2. Depende do tipo de pessoa ou das circunstâncias da pessoa? 10. Acha que as pessoas são capazes de reconhecer se uma febre é malária ou outra doença?     1. Se identificam a malária, isto faz com que as pessoas visitem mais os Médicos Tradicionais do que as US/APEs, ou vice-versa? Porque?     2. Se você reconhece um caso de malária, qual pensa que é a causa? Qual é o atendimento que dá as pessoas? 11. Por vezes recomenda as pessoas a procurar medicamentos na farmácia ou no vendedor informal de fármacos? Se sim, quando? 12. Alguma vez recomenda as pessoas procurar ajuda numa US ou APE? Se sim, quando?   **Perspectivas sobre os serviços e cuidados de saúde modernos**   1. Qual é sua opinião sobre a medicina practicada nas Unidades Sanitárias ou pelos APEs? 2. Você procura serviços e cuidados de saúde nas US/CS ou APEs as vezes? Se sim, para que tipo de desconfortos? 3. Você tem alguma ligação com os serviços e cuidados de saúde? 4. Você considera que é fácil ou difícil para população aceder a serviços e cuidados de saúde? Porquê?    1. Se for difícil, é mais fácil aceder a um Médico Tradicional? Porquê?    2. Isso faz com que as pessoas prefiram visitar os Médicos Tradicionais? 5. Acha que a população tem uma preferência pelos Médicos Tradicionais ou pelas US/CS ou APEs?   **Barreiras e facilitadores da procura de cuidados de febres na US/CS ou num APE**   1. Na sua opinião, quais são as barreiras que a população encontra para ter acesso aos serviços e cuidados de saúde? 2. Na sua opinião, quais são as circunstâncias que ajudam as pessoas ter acesso aos serviços e cuidados de saúde? 3. Qual é a estratégia usada pelo Ministério de saúde para que as pessoas tenham acesso aos serviços e cuidados de saúde? 4. Há outra coisa que está a ser fazer para que as pessoas tenham acesso aos serviços e cuidados de saúde? (ex. transporte, incentivos, comida, horários, etc)   **Recomendações gerais**   1. Na sua opinião, o que tem se melhorar para a população ter acesso aos serviços de saúde com facilidade? Ou está bom como está? |
| --- |

**OBSERVAÇÕES**

NOME DO ENTREVISTADOR: ______________________________

Assinatura: _______________________ CÓDIGO: |__|__|__|__|

S2 Table 6. Guião de discussão focal (GDF) com o grupo da população geral

**INFORMAÇÃO DEMOGRÁFICA DOS PARTICIPANTES**

| **Part.** | **Idade** | **Sexo**  **(F/M)** | **Situação Marital (*1)** | **Nível**  **Escolaridade (*2)** | **Ocupação**  **(*3)** | **Tipo de emprego (*4)** | **Principal forma de sustento da família (*5)** | **Religião**  **(*6)** | **Posto**  **Administrativo (*7)** |
| --- | --- | --- | --- | --- | --- | --- | --- | --- | --- |
| 1 | \|__\|__\| | \|__\| |  | \|__\| |  |  |  |  | \|__\| |
| 2 | \|__\|__\| | \|__\| |  | \|__\| |  |  |  |  | \|__\| |
| 3 | \|__\|__\| | \|__\| |  | \|__\| |  |  |  |  | \|__\| |
| 4 | \|__\|__\| | \|__\| |  | \|__\| |  |  |  |  | \|__\| |
| 5 | \|__\|__\| | \|__\| |  | \|__\| |  |  |  |  | \|__\| |
| 6 | \|__\|__\| | \|__\| |  | \|__\| |  |  |  |  | \|__\| |
| 7 | \|__\|__\| | \|__\| |  | \|__\| |  |  |  |  | \|__\| |
| 8 | \|__\|__\| | \|__\| |  | \|__\| |  |  |  |  | \|__\| |
| 9 | \|__\|__\| | \|__\| |  | \|__\| |  |  |  |  | \|__\| |
| 10 | \|__\|__\| | \|__\| |  | \|__\| |  |  |  |  | \|__\| |
| 11 | \|__\|__\| | \|__\| |  | \|__\| |  |  |  |  | \|__\| |
| 12 | \|__\|__\| | \|__\| |  | \|__\| |  |  |  |  | \|__\| |
| ***1. Situação Marital**: 1-Solteiro/a 2-Casado/a 3-União 4-Viúvo/a 5-Outro (especificar)  ***2. Nível de Escolaridade**: 1-Nenhum 2-Primária 3-Secundária 4-Superior  ***3. Ocupação**: 1- Doméstico 2- Camponês 3- Camponês remunerado 4- Estudante 5-Negociante 6-Serviços 7-Trabalhador da Saúde 8-Outro (esp)  ***4. Tipo de emprego:** 1-Trabalho formal tempo inteiro 2-Trabalho formal tempo parcial 3-Trabalho informal (sem contrato) tempo inteiro 4-Trabalho informal (sem contrato) tempo parcial 5-Trabalho pontual ou esporádico  ***5. Principal forma de sustento da família:** 1-Ingressos do trabalho formal 2-Ingressos do trabalho informal 3-Agricultura 4-Outro  ***6. Religião:** 1-Cristão 2-Islâmico 3-Hindu 4-Animista 5-Ateus 9-Outro(esp)  ***7. Posto Administrativo:** 1-Magude Sede 2-Motaze 3-Panjane 4-Mahele 5-Mapulanguene | | | | | | | | | |

**DADOS DA DGF**

| **Ref. DGF/Ficheiro/Áudio**  **(CARE-SOC-DGF-*NumDGF-mmdd*)** | CARE-SOC-DGF-\|__\|__\|-\|__\|__\|__\|__\| |
| --- | --- |
| **Data** | \|__\|__\|-\|__\|__\|-\|__\|__\|__\|__\| |
| **Local Específico (ex: círculo, escola, etc)** |  |
| **Número Inicial de Participantes** | \|__\|__\| |
| **Número Final de Participantes** | \|__\|__\| |
| **Hora de Inicio da DGF** | \|__\|__\|:\|__\|__\| |
| **Hora do Fim da DGF** | \|__\|__\|:\|__\|__\| |
| **Resultado da DGF** | \|__\| Completa \|__\| Incompleta, razões:_________  Se aplicável, remarcada para: \|__\|__\|-\|__\|__\|-\|__\|__\|__\|__\| |

**CONTEÚDOS DA DISCUSSÃO**

| **Percepções sobre estados de saúde preocupantes e necessidade de procura de cuidados em diferentes formas**   1. Como vocês identificam um estado de saúde anormal ou preocupante em si ou em um membro da sua família? O que lhe indica, normalmente, que você ou um membro da família não está bem? 2. Acham que há alguns desconfortos para os quais é preciso visitar um médico tradicional? 3. Alguma vez procuram medicamentos diretamente a través dum vendedor informal de fármacos? Se sim, para que tipo de desconforto e porquê? 4. Que tipo de desconfortos acha que pode-se tratar em casa? 5. Já ouviram falar de agentes polivalentes (APEs) que existem na comunidade? Na vossa opinião, qual é que acham que é o papel dos APE,s na comunidade? Para que tipo de desconfortos vocês vão a um APE / levam a um membro da família a um APE? 6. Para que tipo de desconfortos vocês vão a Unidade Sanitária / Centro de Saúde / levam a um membro da família a US/CS? 7. Se as vezes visitam um médico tradicional, o que lhes faz escolher entre procurar cuidados num médico tradicional e não numa US/CS ou APE? E ao revés?   **Percepções sobre a febre e as suas causas**   1. Vocês podem identificar a febre? Como se apresenta a febre, normalmente? O que lhes indica que você ou um membro da família tem febre? 2. Quais são as principais causas de febres que vocês conhecem?    1. *Se alguém fala de malária, perguntar sobre a causa da malária e os outros sintomas da malária.* 3. Há febres que têm de ser atendidas de diferentes modos? Se sim, dar exemplos. Está relacionado com a causa da febre?    1. *Se alguém fala de malária, perguntar o que se faz quando tem malária.*    2. *Há febres que só podem ser atendidas pelos médicos tradicionais? E só por APEs ou em US/CS? E só em US/CS?*   **Atitudes em casos de febre**  Quando vocês ou um membro da família sente que têm febre:   1. Que tipo de ajuda vocês procuram primeiro? (ex. cuidados em casa, medicamentos, diagnostico, falar com o líder comunitário, etc) 2. O que lhes leva a procurar ajuda fora de casa? (ex. recomendação dum familiar, não poder sair da cama, outros sintomas além da febre, identificar a febre como malária, etc) 3. Quem é a principal pessoa que toma a decisão sobre o que fazer quando alguém na família tem febre? Em que momento? 4. Qual é a primeira forma de ajuda que vocês procuram fora de casa (ex. APE, US/CS, medico tradicional, chefe comunitário, etc)?    1. Se não for APE/US/CS, o que lhes faz finalmente ir a um APE/US/CS? (se alguma coisa lhes faz ir a APE/US/CS) 5. Quanto tempo acham que demoram, em geral, desde que sente uma febre até se tomar a decisão de ir a um APE/US? (se toma essa decisão) E quanto tempo demoram desde que se toma a decisão até chegar a um APE/US? 6. Têm alguma preferência entre Unidade Sanitária(s) vs APEs para o tratamento das febres? Se sim, a que se deve?   **Barreiras e facilitadores da procura de cuidados de febres na Unidade Sanitária ou Centro de Saúde**   1. Quais são as principais barreiras / dificuldades que vocês encontram quando querem procurar ajuda na US/CS para febre? *Nota: pode haver fatores da sua situação pessoal, fatores relacionados com a US, fatores gerais, outros*.    1. *Algumas destas dificuldades lhes impedem procurar ajuda numa US/CS?* 2. O que lhes facilita a procura de ajuda na US/CS para febre?   **Barreiras e facilitadores da procura de cuidados de febres do APE**   1. Quais são as principais barreiras / dificuldades que vocês encontram quando querem procurar ajuda do APE para febre?    1. *Algumas destas dificuldades lhes impedem procurar ajuda do APE?* 2. O que lhes facilita a procura de ajuda do APE para febre? 3. Têm apoio da família ou na comunidade para cuidar dos membros da família quando vai a US ou APE? Se sim, de quem recebe apoio?   **Perspectivas sobre a qualidade e custo do atendimento nas US / APEs**   1. Quando chegam na US/CS, com febres, qual é o tratamento que vos dão? (Explore mais, se fazem TDR, ou outros exames?) 2. No geral, vocês consideram que recebem um atendimento de boa qualidade na US/CS? Se sim, porque? Se não, porque não? 3. E do APE? Se sim, porque? Se não, porque não? 4. Quais são os principais custos envolvidos no processo de se curar duma febre se vai a US/CS? E a um APE? São acessíveis ou elevados? 5. O custo é um fator que vocês consideram antes de procurar cuidados de saúde?   Quais são os factores que influenciam na tomada de decisão para procurar ajuda na US/APE?   - Distância da US/CS / APE - Transporte - Horário de atendimento - Conhecimentos técnicos ou formação dos trabalhadores de saúde - Tempo de espera ao atendimento - Ferramentas disponíveis na US/APE (diagnostico, medicamento) - A maneira de tratar as pessoas que os trabalhadores de saúde têm - A frequência com que pessoas em casa ficam doentes - O número de pessoas que você há de cuidar - Ter o apoio do seu marido/mulher (econômico ou a nível prático) - Estar familiarizado com o APE ou a Unidade Sanitária/CS - Tempo total que você perde   **Recomendações gerais**   1. Na vossa opinião, o que tem que se melhorar para a população ter acesso aos serviços de saúde com facilidade? Ou está bom como está?   **Caso não tenham identificado a malária na parte de “Percepções sobre a febre e suas causas”: conhecimentos sobre a malária**   1. Na vossa opinião, quando uma pessoa tem febres, arrepios de frio, dores de cabeça e as vezes vómitos, que doença deve ter? 2. O que sabem mais sobre a malária?   • Causa  • Prevenção/ Explorar mais sobre a prevenção  • Tratamento   1. Caso não tenham usado o termo malária, procurar saber se a doença que descreveram tem a ver com malária ou não? 2. Na sua opinião, qual pode ser a causa e o que pode-se fazer para eliminar a malária? 3. Geralmente quando as pessoas têm esses sintomas que se referiram, onde é que recorrem? Incluir também crianças.  - Para aqueles que não vão ao hospital, que outros provedores procuram quando têm febres? |
| --- |

OBSERVAÇÕES _______________________________________________________________________

ASSINATURAS

NOME DO FACILITADOR: _________________________________ Assinatura: _______________________ CÓDIGO: |__|__|__|__|

NOME DO REDACTOR: ____________________________________ Assinatura: _______________________ CÓDIGO: |__|__|__|__|
