# Supplementary material for: Healthcare-seeking behaviour of fever cases in Magude district, southern Mozambique: A qualitative study
Source: PLoS One. 2025 May 29;20(5):e0323117. doi: 10.1371/journal.pone.0323117 (PMC12122035; doi:10.1371/journal.pone.0323117)
Supplement: Attachment — Submitted filename: Response to reviewers.docx [file pone.0323117.s005.docx]

Centro de Investigação da Manhiça

Manhiça District, Maputo Province,

Mozambique

11^th^ August 2024

Dear Editor,

Thank you for reviewing the manuscript “*Understanding healthcare-seeking behaviour of fever cases in Magude district, Southern Mozambique: A qualitative study*” [**PONE-D-24-05377**]. The authors of this manuscript have read the current Instructions for Authors, and agreed to accept the recommedned format. The new manscript version reflects the recommeded format, and we hope it meets PLOS ONE's style requirements. All authors have also read and agreed upon the submitted version of this manuscript. We believe that the new manuscript will now be suitable for publication format in the PLOS ONE journal. We have addressed all comments from acdemic editor and reviewers, as illustred below.

All authors have agreed to the submission of this version.

We appreciate your time and look forward to your response.

Yours sincerely

_______________________________

Carlos Eduardo Cuinhane

Corresponding author

E-mail: [c.cuinhane@hotmail.com](mailto:c.cuinhane@hotmail.com)

1. **Response to Academic Editor**

1. Please ensure that your manuscript meets PLOS ONE's style requirements, including those for file naming. The PLOS ONE style templates can be found at
<https://journals.plos.org/plosone/s/file?id=wjVg/PLOSOne_formatting_sample_main_body.pdf> and
<https://journals.plos.org/plosone/s/file?id=ba62/PLOSOne_formatting_sample_title_authors_affiliations.pdf>

**Answer**: Thank you this recommendation, we have read the guideline for authors, and we formatted the manuscript according to the style recommended.

2. We note that you have indicated that there are restrictions to data sharing for this study. PLOS only allows data to be available upon request if there are legal or ethical restrictions on sharing data publicly. For more information on unacceptable data access restrictions, please see <http://journals.plos.org/plosone/s/data-availability#loc-unacceptable-data-access-restrictions>.

Before we proceed with your manuscript, please address the following prompts:

a) If there are ethical or legal restrictions on sharing a de-identified data set, please explain them in detail (e.g., data contain potentially identifying or sensitive patient information, data are owned by a third-party organization, etc.) and who has imposed them (e.g., a Research Ethics Committee or Institutional Review Board, etc.). Please also provide contact information for a data access committee, ethics committee, or other institutional body to which data requests may be sent.

**Answer**: Yes, there are ethical restriction on sharing a de-identified data set imposed by Ethic Committee, and we which to explain as it follows:

**Data availability statement**

The data of this study were collected under individual-level informed consent after a research protocol was reviewed and approved by CISM’s Institutional Ethic Committee (CIBS-CISM) and the Mozambican Ministry of Health National Bioethics Committee. The informed consent signed by the participants stated that: “data will only be available for the study team”, and the protocol established that all information will be confidential, and no data from data collection forms, nor from audio files will be accessible to anyone outside of CISM. Given this statement approved by the IRBS, data from this study is available upon request to these institutional review boards: CISM’s institutional Ethic Committee ([secretariado.cibis@manhica.net](mailto:secretariado.cibis@manhica.net)) or the Mozambican Ministry of Health National Bioethics Committee ([cnbs.mocambique@gmail.com](mailto:cnbs.mocambique@gmail.com)) for researchers who meet the criteria for access to confidential data.

3. Please include captions for your Supporting Information files at the end of your manuscript, and update any in-text citations to match accordingly. Please see our Supporting Information guidelines for more information: <http://journals.plos.org/plosone/s/supporting-information>.

**Answer**: Thank you for this notification. Actually, we do not have additional supporting information files. We have included captions in the Tables inserted in the text.

2. **Response to reviewers**

**To review 1**

Thank you very for all comments about the manuscript. We have addressed all comments, and we believe they improved the manuscript quality presentation. Bellow, are answers of all questions raised.

METHODS: Check use of commas and full stops when presenting numbers e.g. '63.691 inhabitants and 14.583 households' I would write with a comma, not a full stop. Similarly '2,7km (IQR 1,4-7,9 km)' I would write with a full stop, not a comma. This is perhaps a country-specific preference and should be for the editor to make final decision on.

**Answer**: Thank you for pointing out the error. Based on the recommendations, a full stop was used to write all numbers, while a comma was used to write km.

Some abbreviations used that do not have full description e.g. IQR, NMCP, SSI etc.

**Answer**: Thank you for the observation, the full description of the indicated abbreviations was inserted.

Line 248 - Confronting who?

**Answer**: the sentence was rephrased, and the new sentence is now hopefully clearer: it meant to contrast or compare the transcription with the audio recording to verify that they matched accurately.

RESULTS:

Lines 418 - 436: Seems like a lot of important information is included here about different decision-making categories, but is a bit confusing to understand due to long sentences. Can it perhaps be broken down further, or use numbers to signify the different categories?

**Answer**: The concern about long sentence was addressed in our best capacity. Were broke down some sentences and we rephrased to make it clear.

Line 458: Is women being more 'sensitive' the right word here? Perhaps more aware, or observant, or it needs to be further explained.

**Answer**: Indeed, the word “sensitive” is from participants’ discourse (“sensível” in the original transcript in Portuguese), and thus we have kept the original word from the participants’ discourse in the quoted text. We indeed think it meant that women may be more attentive and observant to children.

Line 549: What kind of health facility? e.g. health centre, clinic, public, private - perhaps more details about the health facilities can be added in the methods. Additionally, perhaps a few lines on the nature of the health system may help the reader better understand the overall health landscape in Mozambique e.g. are the facilities mainly government funded, or privately run? Are there user fees? Insurance schemes?

**Answer**: Health facility here refers to the public health centre managed by the government. In this district there is no private health centre at all. We have described the health service available in this study setting in the method section (study setting) and we provided additional sources for more information. Moreover, in Mozambique all uses of public health services are for almost free. They only pay 1 metical (USD 0,016*) for adult consultation and for free for all children, and 5 meticais (USD0,079) for pills, regardless their social status or level of the health facility. Even though, people who do not have this amount still access health services and pills for free. We have added this in the text.

*Rate: USD 1=63,3 (11/0/2024).

Line 782: Do you need to define what a 'formal' healthcare setting is here? If this includes CHWs, perhaps 'service' is better than 'setting'?

**Answer**: Indeed, the correct word is formal health service. We have corrected it in revised version.

DISCUSSION: Line 953 and 963: The authors could also reference: Virhia J. Contextualising health seeking behaviours for febrile illness: Lived experiences of farmers in northern Tanzania. Health Place. 2022 Jan;73:102710. doi: 10.1016/j.healthplace.2021.102710. Epub 2021 Nov 19. PMID: 34801785 here also in addition to reference 33.

**Answer**: Thank you for this suggestion. We have included Virhia in the discussion section of the revised manuscript.

Overall, this is a well thought out and implemented research study. The findings are informative and will contribute to the wider literature on health seeking across various contexts. The only key thing I would suggest to add is perhaps providing a diagram or table or description of the overall health landscape in Mozambique identifying who and what the key health services are, whether they are formal/informal/public/private etc. There are many different services and actors mentioned in the study so it may just be useful to have some way to summarise them at the beginning of the results section. See Davis et al, 2021: “If You Do Not Take the Medicine and Complete the Dose…It Could Cause You More Trouble”: Bringing Awareness, Local Knowledge and Experience into Antimicrobial Stewardship in Tanzania - Table 2 (animal health) for a suggestion on how this could be done.

**Answer**: Thank you for this suggestion. We think that it is a very interesting form of presenting all available health services, but we are lacking data to map all health services in Mozambique, particularly informal ones. The study did map all health services as the article of Davis et al. (2021) did, but rather record only those services emerging from the discourse of the participants. Therefore, we cannot provide a similar table or diagram because we risk to excluded other unknown informal services that did not come out from the data. Moreover, as it was early explained, in Mozambique there is only Public and private health facilities. In urban area there are public and private health services, while in rural areas there are mainly public health services, which we designate health facilities and community health workers. In the study setting there are only 2 formal health services: public health facilities and community health workers. In the text, we have pointed the reader towards a paper on the census of the study district where all public health facilities and community health workers were mapped and described further.

Please check minor spelling errors throughout.

**Answer**: Thank you very much for warming about it. We have revised the spelling throughout the text.

REFERENCES: Reference 33 should be spelled 'Virhia' not 'Verhia'

**Answer**: Thank you, error corrected.

**To reviewer 2**

Thank you very for all comments about the manuscript. We have addressed all comments, and we believe they improved the manuscript quality presentation. Bellow, are answers of all questions raised.

Reviewer #2: Thank you for the change to review this paper. The study’s findings have implication for Mozambique’s malaria elimination fight. However, the manuscript will benefit from major revisions. Find below my comments:
Title & Abstract

**1.Delete “Understanding” from the title.**

**Answer**: the new title does not contain the word “understanding”.

2.The Abstract does not present the research problem necessitating the study. “Qualitative approach” is not a study design. The authors should state the exact study design the used for the study. Data collection tool(s) and data analysis tool should be stated. Revise your conclusion statement to provide an overall implication of the findings and at least a recommendation.

**Answer**: We have revised the abstract to meet the recommendations. However, it was not possible to include all the recommended elements due to the limited word count of 300 words as per PLOS ONE Journal guidelines.

Introduction
3.The introduction lacks global and regional context. What is the malaria/fever situation globally, regionally, and nationally? What at are the key challenges to malaria/fever globally, regionally, and nationally that have been linked to poor healthcare seeking behaviour? Why is healthcare seeking behaviour in fever cases important?

**Answer**: Further regional and national context on the fever and malaria situation, challenges associated and the implication of care seeking on malaria has been incorporated in the text.

4.Again, the research problem is unclear and not strongly drafted. The authors need to highlight the impact of poor healthcare seeking behaviour on fever/malaria fight in the country and the district. Why is this district peculiar?

**Answer**: The implication of poor healthcare seeking behaviour for fever on the malaria efforts in this particular district that is unique in that it is undergoing a malaria elimination pilot project has been strengthened in the text. It is important to mention that this study was designed under a malaria elimination research project, and in the text, the reader is pointed towards a number of scientific papers that have been published about it for context, which may answer further questions the reader may have, including:

- Galatas B, Saúte F, Martí-Soler H, Nhamussua L, Simone W, et al. A multiple program for malaria elimination in southern Mozambique (the Magude project): A before-after study. PLoS ONE. 2020; 17(8): e1003227. <https://doi.org/10.1371/journal.pmed.1003227>

- Galatas B, Nhacolo A, Marti-Soler H, Munguambe K, Jaimise E, Guinovart C, et al. Demographic and health community-based surveys to inform a malaria elimination project in Magude district, Southern Mozambique. BMJ Open. 2020; 10: e033985. <https://doi.org/10.1136/bmjopen-2019-033985>.

- Galatas B, Nhamtumbo H, Soares R, Djive H, Murato I, Simone W, et al. Community acceptability to antimalarial mass drug administration in Magude district, southern Mozambique: A mixed method study. PLoS ONE. 2021; 16(3): e0249080. <https://doi.org/10.1371/journal.pone.0249080>

- Portugaliza HP, Galatas B, Nhantumbo H, Djive H, Murato I, Saúte F, Aide P, Pell C, Muguambe K. Examing community perceptions of malaria to inform eliminations efforts in southern Mozambique: a qualitative study. 2019; 18: 232. <https://doi.org/101186/s12936-019-2867-y>

- Aide P, Candrinho B, Galatas B, Munguambe K, Guinovart C, Luis F, et al. Setting the scene and generating evidence for malaria elimination in Southern Mozambique. Malaria Journal. 2019; 18:190. https//doi.org/10.1186/s12936-019-2832-9 PMID: 31170984.

5.Being a qualitative study, this study would have benefitted from a conceptual/theoretical framework. In the results and discussion sections, the authors make references to “individual” “community” and “health facility” level factors. This suggests that the SEM/HUM would best fit the study and its findings in context.

**Answer**: We do appreciate this suggestion, we have specified the theoretical orientation: the social ecological model was added as suggested.

**Methods**

6.The report should follow the COREQ guidelines for reporting qualitative studies.

**Answer**: we checked the COREQ guidelines for reporting qualitative studies, which was attached within the submission of this manuscript, and we revised this manuscript accordingly. All aspect mentioned in this guideline are inserted in the method. However, we understand that these 32-item check list do not represent the recommend structure of the manuscript as indicated by PLOS ONE. Therefore, the report followed the recommended structure, and considered logically the COREQ guidelines.

7.Revise sentence 1 of paragraph 2 of Study site. The authors use commas instead of points in decimals. These should be rectified.

**Answer**: Thank you, this sentence was revised, and points were used instead of commas.

8.The study design is not stated aside from the authors stating that the approach as qualitative nested in a mixed methods pilot study. Also, why the study design was chosen should be justified. The last sentence is not supposed to be in the study design sub-section but in the procedures.

**Answer**: Thank you for the observation, the study design is now specified in the new revised version and the justification was provided.

9.On the population, the authors state two categories of mothers of children under 15 years. This does not feature in the results and discussions as such there is not need for this designation in this sub-section. The justification for the population chosen for this study should be explained in this sub-section. What were the inclusion and exclusion criteria?

**Answer**: We appreciate the review suggestion about eliminating one of the categories of “mothers”, but actually the study does not use “mother” but women with or without decision-making who were mothers of children under 15 years old, as highlighted in the table of sample size. These two categories were important to analyse healthcare seeking for fever cases as discussed in one of the main themes “health-seeking behaviour”, as the decision-making power highly affected the promptness with which they sought care at formal health services, especially for their children, as stated in the text. In addition, a justification for choosing the included categories was provided. The inclusion criteria were presented Table 1, but as suggested by the reviewer, we deleted the table and we added the inclusion and exclusion criteria in the text.

10.Rather than the sub-section Recruitment procedures, there should be two subsections, namely; Sampling (where you describe how the different participants were selected and included in the study) and Procedures (which details the data collection procedures including the data collection tools, approaches, and process of data collection). Authors should delete Tables 1 and 2 as they are of little importance if they are able to provide better details of the participants in the Population and Sampling subsections.

**Answer**: We followed most of the recommendations suggested as highlighted in the revised manuscript. However, we think that table 2 is important because it highlight the sample size within different groups of the community. We think that transforming this table in words could create misunderstanding.

11.The meaning of some abbreviations/acronyms used have not be provided in the text. For example, SSI, FGD.

**Answer**: Thank you, the meaning of all abbreviation/acronyms was provided in the new revised manuscript.

12.How long each interview and FGD took should be stated.

**Answer**: the duration of interviews and FGD was proved in the last version.

13.In the Abstract, the authors stated that the analysis was thematic. However, in the Data analysis subsection, they state that they used content analysis approach. This should be consolidated and resolved.

**Answer**: A thematic analysis was used to analyse the data. We have corrected and harmonized the indicated discrepancy in revised version.

**Results**
14.The first sentence of the sociodemographic characteristics should be sent to the methods section. This is not a result. I also think there should be separate tables for IDI participants and FGD participants.

**Answer**: We appreciate the suggestion, and we have moved the sociodemographic characteristics of the participants to “sampling” section. The two groups (SSI and FGD) were presented separately.

15.There should a thematic table that summarise the key themes and subthemes identified in the analysis.

**Answer**: We appreciate the suggestion, but think that a table would represent the content of the result section, which seems not to be recommended by the Journal guidelines. However, to facilitate the reader we have now described the main themes and its sub-themes in text. We hope this will provide a clear understanding of themes and sub-themes emerging from the data.

16.Separate the subsection for Facilitators and Barriers. This will allow for you to subsection the different levels of factors/barriers and also make it easier for readers’ reading and understanding.

**Answer**: Thank you, the subsection for facilitators and barriers are now separated as shown in the revised version.

Discussion
17.The first paragraph should restate the specific objectives for the study and the key findings for each which the section discusses.

**Answer**: The specific objectives and the respective study finding are now integrated in the discussion.

18.Restructure the discussion to be more coherent and logically flowing following the findings of the specific objectives.

**Answer**: Thank you, the new revised version presents a restructured discussion, following the logic of the finding of the specific objectives.

19.The discussion section is deficient in literature comparison, proferring explanations for the study’s observations, and the meaning as well as implications of the findings. Please revise.

**Answer**: The discussion was revised and the comparison with the literature was added accordingly.

Conclusion
20.This section needs revision. The authors only offered to summarise the findings of the study which is not the purpose of this section. Here, the authors should discuss the overall implication of their major findings to policy, practice, and research and based on these, appropriate provide recommendations.

**Answer**: The section was revised and the conclusion and main recommendations were provided. However, to prevent repetition and pulling discussion to conclusion, authors preferred to discuss the implication of each result in the discussion section and keep main recommendations only in the conclusion to keep it clear and brief.
