# Supplementary material for: Healthcare-seeking behaviour of fever cases in Magude district, southern Mozambique: A qualitative study
Source: PLoS One. 2025 May 29;20(5):e0323117. doi: 10.1371/journal.pone.0323117 (PMC12122035; doi:10.1371/journal.pone.0323117)
Supplement: Attachment — Submitted filename: Response_to_reviewers_auresp_2.docx [file pone.0323117.s006.docx]

1. **Response to Editor**

We have reviewed the reference list to ensure that it is complete and correct. None of the papers cited have been retracted. Some editorial changes have been made for typos found in some of the references’ names.

Please note we have specified that the two first authors of the list are co-first authors of the paper for having contributed equally to this work. Please let us know if we should take any further steps to ensure this is reflected in the publication.

2. **Response to reviewers**

Abstract:
1. Although I understand the concern of the author(s) about the word count for the abstract. However, I still find the abstract deficient. The abstract should clearly summarise why the study, what methods were done, what findings were obtained, and what is the implication of the findings. As such, please revise to ensure the research problem is stated. For instance, was the data analysis done manual or a software was used? This should be stated.

**Answer:** Thank you for guiding us on how to make the abstract more impactful. We have revised the abstract to make sure the elements recommended are presented, including the research question necessitating the study. We have also included the methods used, the software used for data management, as well as a summary of the main findings and their implications and recommendations.

Introduction:
2. This has been improved significantly. However, lines 143-146 are hanging. A single sentence cannot pass as a paragraph. The authors should revise.

**Answer:** Thank you very much for this observation. The issue has been addressed and the lines are no longer hanging, and language has been revised in the introduction.

Methods:
3. On the population, my previous comment was not to delete a category of mothers. The concern raised was that since the authors argue that this categorization was significant, it expected that it shows in the results and discussions. However, the authors were silent about how this categorization’s peculiar background presents in the findings and the discussion, making this categorization obsolete. Please let this reflect in the details of the quotes and highlight this is in the discussion if relevant findings support this.

**Answer:** Thank you for the observation. We have reviewed the representation of these categories in the results and discussion sections and made some relevant changes to present the specific idiosyncrasies associated to health care seeking behaviors in families where women have decision making powers in contrast to those where they don’t, showing that women with decision making power promptly seek care for children. We have realized that despite including relevant quotes from women with decision-making power, community leaders (which is the group of man who specifically stated women in their households hold the decision-making power in relation to child care), and women without decision-making power, we needed to make the relevance of the quotes explicit in the text in order for this difference to be better displayed. We have now done so within results, in the section on decision-making processes and timing to seek care (lines 483-517). Because the decision-making power held by women, who are the main caretakers of children in this community, affects the promptness with which care is sought in this study and other studies, this has been discussed in the discussion in lines 1058-1093. It is important to explain that the “category of mother” was not labelled as such in this study, but a characteristic of a category designated “women with or without decision making”, who are mothers of children under 15 years old, as presented in Table 1. Based on this understanding, we have reviewed the representation of these categories (women with decision making and women without decision-making) in the results and discussion sections.

Results:
4. I believe socio-demographic information of the study participants should be presented in the results section as they were not predetermined and considered part of the results.

**Answer:** We have welcomed the observation and moved the socio-demographic information to the results section as suggested.

5. I still insist that authors should include a thematic table of the findings. This is not a journal requirement but a good qualitative study practice. It gives a snapshot of the findings and help readers understand the organization and flow of the themes and their subthemes. Please provide this.

**Answer:** We agree that this will help he reader have a snapshot of the main themes and sub-themes in the study and thus a thematic table of findings has now been included in the results section, thank you for the suggestion.

6. Rather than quoting even the researcher’s questions in the quotes, it is best practice to only quote that of the participant and add any context/explanation in parenthesis. For instance, lines 865-872 should best be presented as: “Today, I have a cough. It started yesterday. …I made syrup. …(no need to go to health facility because) it is still not strong. …Yes (it is able to treat oneself at home).”

**Answer:** Thank you very much for pointing this out. We have corrected the presentation of quotes to show the participants’ answers only in quotes that previously contained researcher’s questions (lines 386-390; 516-517; 744-747; 810-812).

Discussion:
7. This has improved a lot. However, it is not enough just stating that a finding is similar or different from that of previous studies. Authors should offer at least a possible explanation for difference in findings.

**Answer:** Thank you, this suggestion enrichens the relevance of the study in its specific context. We have now provided possible explanations for contrasting findings as well as for some findings aligned with other studies, in lines 1027-1032, 1051-1056, 1116-1119, and 1169-1179 of the discussion.

8. The subsection limitations should instead be Strengths and Limitations to also espouse the strengths of the study and not only the limitations.

**Answer:** Thank you very much for pointing this out. We have course corrected and included study strengths in lines 1229-1238 as part of the “Strengths and limitations” section.
